# Ubl4A is critical for mitochondrial fusion process under nutrient deprivation stress

**DOI:** 10.1371/journal.pone.0242700

**Published:** 2020-11-19

**Authors:** Huaiyuan Zhang, Yu Zhao, Qi Yao, Zijing Ye, Adriana Mañas, Jialing Xiang

**Affiliations:** Department of Biology, Illinois Institute of Technology, Chicago, IL, United States of America; University College London, UNITED KINGDOM

## Abstract

Mitochondrial fusion and fission are dynamic processes regulated by the cellular microenvironment. Under nutrient starvation conditions, mitochondrial fusion is strengthened for energy conservation. We have previously shown that newborns of Ubl4A-deficient mice were more sensitive to starvation stress with a higher rate of mortality than their wild-type littermates. Ubl4A binds with the actin-related protein Arp2/3 complex to synergize the actin branching process. Here, we showed that deficiency in Ubl4A resulted in mitochondrial fragmentation and apoptosis. A defect in the fusion process was the main cause of the mitochondrial fragmentation and resulted from a shortage of primed Arp2/3 complex pool around the mitochondria in the Ubl4A-deficient cells compared to the wild-type cells. As a result, the mitochondrial fusion process was not undertaken quickly enough to sustain starvation stress-induced cell death. Consequently, fragmented mitochondria lost their membrane integrity and ROS was accumulated to trigger caspase 9-dependent apoptosis before autophagic rescue. Furthermore, the wild-type Ubl4A, but not the Arp2/3-binding deficient mutant, could rescue the starvation-induced mitochondrial fragmentation phenotype. These results suggest that Ubl4A promotes the mitochondrial fusion process via Arp2/3 complex during the initial response to nutrient deprivation for cell survival.

## Introduction

Mitochondrial fusion and fission are dynamic processes in response to environmental changes [1]. Stress conditions and limited energy supplies can significantly cause mitochondrial morphological changes [2–4]. Under nutrient deprivation conditions, such as during birth, mitochondria initially condense and elongate to improve energy conservation and avoid autophagic degradation [5,6]. The elongation process is achieved by promoting the fusion process through the outer mitochondrial membrane proteins mitofusion 1 (Mfn1) and mitofusion 2 (Mfn2) [7] and by inhibiting the fission process controlled by GTPase dynamin-related protein 1 (Drp1) [8–10]. Dysregulation of mitochondrial homeostasis at this stage often leads to the fragmentation of mitochondria and reduced ATP production, and consequently, the accumulation of reactive oxygen species (ROS), and cell death [10–14]. Autophagy is another important protective mechanism initiated during starvation. Fragmented mitochondria can trigger mitophagy for cell survival [15–17]. However, the timing is a critical factor in the determination of cell fate. If the mitochondria-mediated caspase cascade is activated before autophagosomal formation, apoptosis may be initiated, and cell death cannot be avoided.

The mitochondrial fission and fusion processes, as well as mitophagy, are controlled by the actin-branching network [18–21]. The buildup of actin branches depends on the actin-related protein Arp2/3 complex, which recruits daughter F-actin to the mother actin microfilament to form Y-shaped branches [22–25]. In yeast, the Arp2/3 complexes are localized on the surface of mitochondria and regulate mitochondrial mobility [26]. Mutations in the Arp2/3 complex subunits result in defects in mitochondrial morphology and inhibition of mitochondrial movement in budding yeast [27–29]. The relationship between Arp2/3-actin and mitochondrial fission has been well documented in mammalian cells. Actin cyclic assembly around mitochondria promotes mitochondrial fission; therefore, inhibition of actin assembly can release mitochondria for fusion to restore a fission-fusion balance [20,30–32]. Upon the treatment of mitochondrial uncoupler, such as FCCP, a transient assembly of F-actin can be observed on the outer mitochondrial membrane to facilitate mitochondrial fission, such process can be blocked by knocking down actin regulatory proteins such as cortactin and the Arp2/3 complex [30]. The underlying mechanism of the mitochondrial fission is executed through Drp1. F-actin recruits Drp1 proteins to the outer mitochondrial membrane where they form a ring-like structure at the fission point to separate the mitochondria [31–36]. However, phosphorylation of Drp1 (S637) leads to the dissociation of Drp1 from mitochondria, thereby mitigating the mitochondrial fission process and facilitating the fusion process. Interestingly, the outcomes of actin organization can also depend on the cell type and microenvironment. For example, treatment with the actin inhibitor Latrunculin B caused mitochondrial elongation in U2OS osteosarcoma cells but disabled mitochondrial mobility in cultured neurons [31,32,37,38]. The functional roles of the Arp2/3-actin network on mitochondria under starvation conditions remain to be explored.

Ubl4A is a small protein composed of 157 amino acids. Although it belongs to the ubiquitin-like family, Ubl4A has no ubiquitination activity [39]. Instead, this protein has diverse functions, from chaperoning nascent synthesized proteins to the ER, promoting anti-tumorigenesis, potentiating autoimmune diseases, to augmenting innate immune response through NFκB signaling pathway [40–48]. We have shown that Ubl4A can directly interact with the Arp2/3 complex to promote actin branching [49]. Upon starvation stimulation, such Arp2/3-actin branches can serve as “bridges” for the translocation of certain molecules, such as protein kinase B (Akt), from the cytosol to the plasma membrane for glucose metabolism and cell survival [49]. Knocking out Ubl4A disabled the translocation of Akt and led to defects in glycogen storage in neonatal livers, consequently resulting in a high mortality of newborns [49]. In this study, we show that Ubl4A-Arp2/3 is also critical for mitochondrial fusion under starvation conditions. Ubl4A deficiency or Arp2/3 inhibition can significantly impair the mitochondrial fusion ability. Ubl4A-deficient cells contain less primed Arp2/3 serving as a “ready-to-go” pool around mitochondria. As a result, upon starvation insult, mitochondria are unable to fuse rapidly, soon become fragmented, lose their integrity, and trigger mitochondria-mediated apoptosis at an early stage of starvation. Wild-type Ubl4A, but not the Arp2/3-binding deficient mutant, can rescue the mitochondrial fragmentation phenotype. These results suggest that Ubl4A acts through the Arp2/3 complex to ensure mitochondrial fusion quickly enough for cell survival in the initial response to starvation stress.

## Materials and methods

The animal study was approved by the Illinois Institute of Technology IACUC.

### Reagents

Dulbecco’s modified Eagle’s medium (DMEM), phosphate-buffered saline (PBS), and Hank’s balanced salt solution with Ca^2+^/Mg^2+^ (HBSS) were purchased from Corning Inc (Corning, NY). Fetal bovine serum (FBS) was obtained from Gemini Bio-Products (West Sacramento, CA). SiRNA for GFP (control, sc-45924) was obtained from Santa Cruz Biotechnology (Dallas, TX). ON-TARGETplus Mouse ArpC2 siRNA (L-043464, SMARTpool of 4 with anti-sense sequences: 5’-GGGACUAUCUGCACUACCAtt-3’, 5’-GCCUAUAUUCAUACACGAAtt-3’, 5’-AGGAAGCGC-UGUCGACCGAtt-3’, 5’-GGUAAUGAGUUGCAGGUAAtt-3’) and ON-TARGETplus Mouse Actr3 (Arp3, L-046642, SMARTpool of 4 with anti-sense sequences: 5’-GAAGAGAGCU-AAGACGAUUtt-3’, 5’-AAGCAGUGAAGGAACGCUAtt-3’, 5’-GCUGACGGGUACAGUAAUAtt-3’, 5’-GAGUCAACGCCAUCUCAAAtt-3’) were obtained from Horizon Discovery. Ethidium homodimer-1 (EthD-1) was purchased from Molecular Probes (Eugene, OR). For immunofluorescence staining, mouse anti-Tom20 (1:200) (AB56783, Abcam), anti-Mfn1 (1:200) (AB57602, Abcam), rabbit anti-Tom20 (1:200) (SC-11415, Santa Cruz Biotechnology), and anti-ArpC2 (1:200) (07–227, Millipore) antibodies were used. For immunoblotting, mouse anti-actin (1:3000) (MAB1501R, Millipore), anti-caspase 9 (1:800) (9508S, Cell Signaling), anti-Drp1 (1:1000) (14647S, Cell Signaling), rabbit anti-p-Drp1 (Ser616) (1:1000) (3455S, Cell Signaling) and anti-p-Drp1 (S637) (1:1000) (6319S, Cell Signaling) were used. The secondary antibodies Alexa Fluor 488, Alexa Fluor 596 and Alexa Fluor 647 from Molecular Probes were used. NucView^®^ 530 Caspase-3 substrate was purchased from Biotium (Fremont, CA). Tetramethylrhodamine ethyl ester perchlorate (TMRE), 2’,7’-dichlorofluorescin diacetate (H2DCF), CK-666, butylated hydroxyanisole (BHA), chloroquine (CQ), phalloidin and all other chemicals used in the study were obtained from Millipore Sigma (Burlington, MA).

### Mice and MEF culture

All animal studies were conducted according to the guidelines approved by the Institutional Animal Care and Use Committee (IACUC) at Illinois Institution of Technology. Mouse embryonic fibroblasts (MEFs) derived from *Ubl4A*-deficient mice (KO) and wild-type (WT) littermates were collected as previously described [49]. The cells were maintained in DMEM supplemented with 10% FBS at 37°C with 5% CO_2_ and passaged every two days. For nutrient deprivation, MEFs were washed two times with HBSS, and then incubated with HBSS for 2 h unless as indicated otherwise. To ensure starvation conditions work well, parallel WT and KO dishes were treated under the same conditions along each experiment for 16 hours for analysis of cell death between WT and KO cells for the consistency of all experiments.

### Immunofluorescence microscopy

WT and KO MEFs cells were cultured on cover slips coated with 0.1% gelatin in 6-well plates prior to the experiments. After starvation, the MEFs were fixed with 4% paraformaldehyde (PFA) in PBS for 15 min and blocked with 5% BSA in PBS containing 0.5% Triton X-100 for 20 min at room temperature and incubated overnight at 4°C with the primary antibody indicated in the figure legends. The cells were then washed with PBS for 3 times and incubated with secondary antibody for 1 h at room temperature. The images were obtained using a BZ-X700 deconvolutional microscope (Keyence, Osaka, Japan) or a CSU-W1 spinning disc confocal microscope (Nikon, Tokyo, Japan) with 2 Hamamatsu Flash 4 cameras at the Northwestern University Center for Advanced Microscopy facility, and data were processed with ImageJ 1.51w software. For hepatic mitochondria staining, neonatal livers from both WT and KO pups were harvested shortly after birth (~6 h) and stained with anti-Tom20 antibody. And images were acquired with CSU-W1 spinning disc confocal microscope as described above. For mitochondrial length quantification, images from both WT and KO (n = 15 for each group) with mitochondrial staining were analyzed for average mitochondrial length in ImageJ with a marco kindly provided by the Microscopy Core Facility at the University of Chicago. Intact whole mitochondrial length from 5 random areas in each image were manually measured to further validate the results by the marco. For mitochondrial quantification of elongation and fragmentation, both WT and KO cell images (n = 25 for each group, unless specified elsewhere) were subjected to a blind-scoring based on the mitochondrial morphology by 3 different viewers. The percentage for each sub-type of mitochondria with different categories (fragmented, intermedium or elongated) was calculated based on the scoring results.

### Cell death assay and caspase 3 activity assay

For the cell death assay, WT and KO MEFs were cultured in 6-well plates to a confluency of approximately 80% prior to the experiment. Cells were washed and starved in HBSS with or without inhibitors as indicated in the figure legends for 16 h. After starvation, the cells were incubated with 1 μM EthD-1 at 37°C with 5% CO_2_ for 15 min. Both floating cells and attached cells were collected after incubation, and the samples were resuspended in PBS containing 1% EDTA for analysis with an Accuri C6 Plus flow cytometer (Becton Dickinson, Franklin Lakes, NJ). The percentage of dead cells was calculated by dividing the number of cells with red fluorescence by the total number of cells in the population. For the caspase 3 activity assay, cells were incubated with NucView^®^ 530 Caspase-3 substrate for 30 min, followed by analysis with flow cytometer as described above.

### Mitochondrial transmembrane potential and ROS level assays

WT and KO MEFs were treated with 1 μM TMRE or 10 μM H2DCF, respectively, for 20 min prior to the harvest of cells starved for different times as indicated in the text. The samples were analyzed with an Attune NxT flow cytometer (Invitrogen, Carlsbad, CA). For the mitochondrial transmembrane potential assay, the total cells were classified into three different categories according to the TMRE fluorescence intensities: cell with high, medium, or low mitochondrial transmembrane potential. The percentage of cells in each category was calculated individually, and the ratio of the cells with low mitochondrial transmembrane potential was compared between the WT and KO MEFs. For the ROS assay, the fold changes in H2DCF-positive WT and KO cells relative to the base line was calculated individually, and the fold changes for the two cell lines were compared.

### Immunoblotting

Harvested MEFs were lysed in NP-40 buffer containing 145 mM NaCl, 5 mM MgCl_2_, 1 mM EGTA, 0.25% NP-40, 20 mM HEPES at pH 7.4, 10 μg/mL aprotinin, 10 μg/mL leupeptin and 10 mM DTT for 30 min at 4°C. Proteins were separated by SDS-PAGE, transferred onto a PVDF membrane, and blocked with 3% nonfat dry milk or 5% BSA for 1 h at room temperature, and then incubated overnight with the appropriate primary antibodies at 4°C. Horseradish peroxidase (HRP)-conjugated goat anti-mouse or anti-rabbit IgG was used as the secondary antibody. The immunocomplexes were visualized using Pierce^TM^ ECL Western blotting substrate (Thermo Fisher Scientific, Waltham, MA), according to the manufacturer’s protocol.

### DNA constructs and transfection

Plasmids encoding GFP-tagged mouse Ubl4A WT and mutant (D122A, aspartic acid to alanine at position 122) were constructed as described previously [50]. DsRed2-Mito-7 and mEGFP-Lifeact-7 were gifts from Michael Davidson (Addgene plasmids # 55838 and # 54610, respectively). WT and KO MEFs were transfected chemically with Lipofectamine^TM^ 3000 transfection reagent (Invitrogen, Carlsbad, CA) according to the manufacturer’s protocol. Briefly, cells were cultured on gelatin coated coverslips in a 6-well plate and allowed to reach a confluency around 60% prior to the experiment. A total of ~2.5 μg DNA was transfected into cells, and after 24 h, the cells were imaged with a deconvolutional or confocal microscope as mentioned as described. For siRNA transfection, each individual siRNAs were transfected with Lipofectamine^TM^ 3000 following the manufacturer’s protocol. Briefly, cells were seeded one day prior to siRNA transfection and reached a confluency ~50% the next day. On the day of experiment, 1 μg of each siRNA pool was diluted with 125 μL Opti-MEM (Gibco, NY) containing 5 μL of P3000 reagent, then combined with another 125 μL Opti-MEM containing 4 μL of Lipofectamine 3000 reagent. The mixture was incubated at room temperature for 20 min to obtain a better transfection efficiency. The whole mixture was then added onto pre-washed cells and incubated at 37°C with 5% CO_2_ for 1 h, followed by adding DMEM with 10% FBS to a final volume of 1.5 mL. The cells were cultured for 24 h and subjected to either immunofluorescence staining or immunoblotting on the next day.

### Time-lapse video

WT and KO MEFs were co-transfected with DsRed2-Mito-7 and mEGFP-Lifeact-7 in 35 mm glass bottom microwell dishes (MatTek Corporation, Ashland, MA). The cells were starved in HBSS for 1.5 h before capturing the time-lapse movies in a heated chamber with a CSU-W1 spinning disc confocal microscope (Nikon, Tokyo, Japan) at the Northwestern University Center for Advanced Microscopy facility. Images were obtained at intervals of 15 sec for a total time of 6 min. The collected data were processed with ImageJ 1.51w software.

### Transmission electron microscopy

Briefly, livers harvested from both WT and Ubl4A KO newborn pups (~6 h after birth) were fixed overnight in 0.1 M sodium cacodylate buffer containing 4% PFA. After fixation, the tissues were incubated in 0.1 M sodium cacodylate buffer with 1% osmium tetroxide for 1 h, followed by staining with 1% uranyl acetate in maleate buffer for 1 h. The samples were further processed and subjected to transmission electron microscopy at the Advanced Electron Microscopy Facility at the University of Chicago with an FEI Tecnai F30 electron microscope.

### Statistics

The data were analyzed using ImageJ 1.51w software. Parametrical statistical analysis was conducted with Student’s *t*-test or one-way ANOVA followed by Tukey’s test using R version 3.2.4. A non-parametric statistical analysis was performed with one-way ANOVA followed by Mann-Whitney U test using GraphPad Prism version 7.03. p values < 0.05 were considered significant: * < 0.05, ** < 0.01, and *** < 0.001. All values represent the means ± SD. For all statistical analysis, experiments had been performed independently for at least three times.

## Results

### Sensitivity of Ubl4A-deficient cells to starvation-induced death is associated with mitochondrial fragmentation

**Fig 1:**
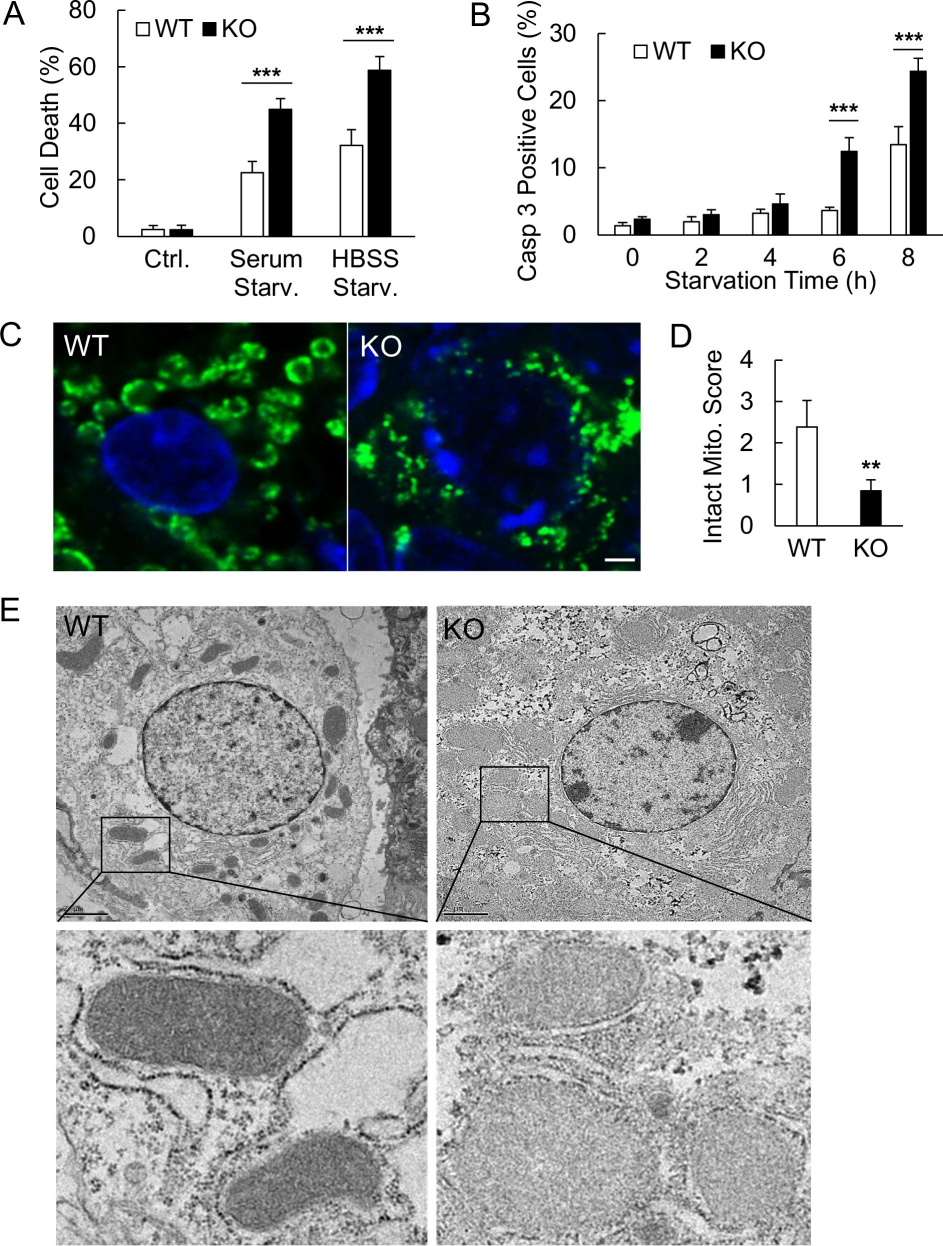
Sensitivity of Ubl4A-deficient cells to starvation-induced death is associated with mitochondrial fragmentation. (A) Cell death assay of wild-type (WT) and Ubl4A-deficient (KO) mouse embryonic fibroblasts (MEFs) in serum free or in HBSS medium for starvation (Starv.) for 16 h. Cell death was measured by the cellular membrane permeabilization to ethidium homodimer-1 (EthD-1). (B) Caspase 3 activation assay of the WT and KO MEFs under HBSS starvation at the indicated time points using NucView^®^ 530 Caspase-3 substrate. (C) Liver tissue sections from WT and Ubl4A KO neonates (< 6 h after birth) immune-stained with anti-Tom 20 antibody. Bar, 20 μm. (D) Quantitative analysis of liver mitochondrial integrity by matrix score. Tiled images with similar number of nuclei from each whole tissue section were manually assigned score based on the integrity of the mitochondria. The integrity of mitochondria is scored as 0 (fragmented), 1 (mostly fragmented), 2 (intermedium), 3 (mostly intact), or 4 (intact); n = 5 for WT and n = 17 for KO neonates. ** p < 0.01. (E) Transmission electron microscopy of the ultrastructure of the mitochondria in the WT and KO neonatal liver hepatocytes (~6 h after birth). Bar, 2 μm.

We have previously shown that Ubl4A is critical for starvation-induced death both *in vivo* and *ex vivo* [49,50]. To determine the nature of the starvation-induced death in Ubl4A-deficient cells, we subjected both wild-type (WT) and *Ubl4A*-/- (KO) mouse embryo fibroblasts (MEFs) to two different types of nutrient deprivation (Fig 1A). We found that the Ubl4A KO cells were more sensitive to both starvation conditions. Activation of caspase 3 in the Ubl4A KO cells could be detected as early as 6 h upon starvation, significantly earlier than that in the WT cells (8 h) (Fig 1B). Independently, we previously showed that Ubl4A KO neonates (shortly after birth, ~6 h) had significant less glycogen storage in hepatocytes and had a higher mortality rate than the WT littermates [49]. Further looking into the hepatocytes, we found that the mitochondria in the WT newborn manifested the typical donut shape with a well-defined boundary, but majority of the mitochondria in the KO hepatocytes were fragmented and disorganized (Fig 1C and 1D). To confirm this finding, we also examined the hepatic mitochondria from neonatal mice using electronic microscopy (EM). The ultrastructure observed using EM also indicated that the mitochondria in the WT hepatocytes were well defined and condensed as expected for energy conservation at an early stage after birth [51]. Intriguingly, we did not observe the donut shaped mitochondria in the EM images as seen in the immunostained images in the neonatal hepatocytes. However, the mitochondria in the KO hepatocytes were deformed and swollen with light density (Fig 1E), which is consistent with our observation of immunostained mitochondria form the KO hepatocytes (Fig 1C). In consideration of the fundamental roles of mitochondria in cell survival, we hypothesized that such deformed mitochondria might associate with starvation-induced cell death.

**Fig 2:**
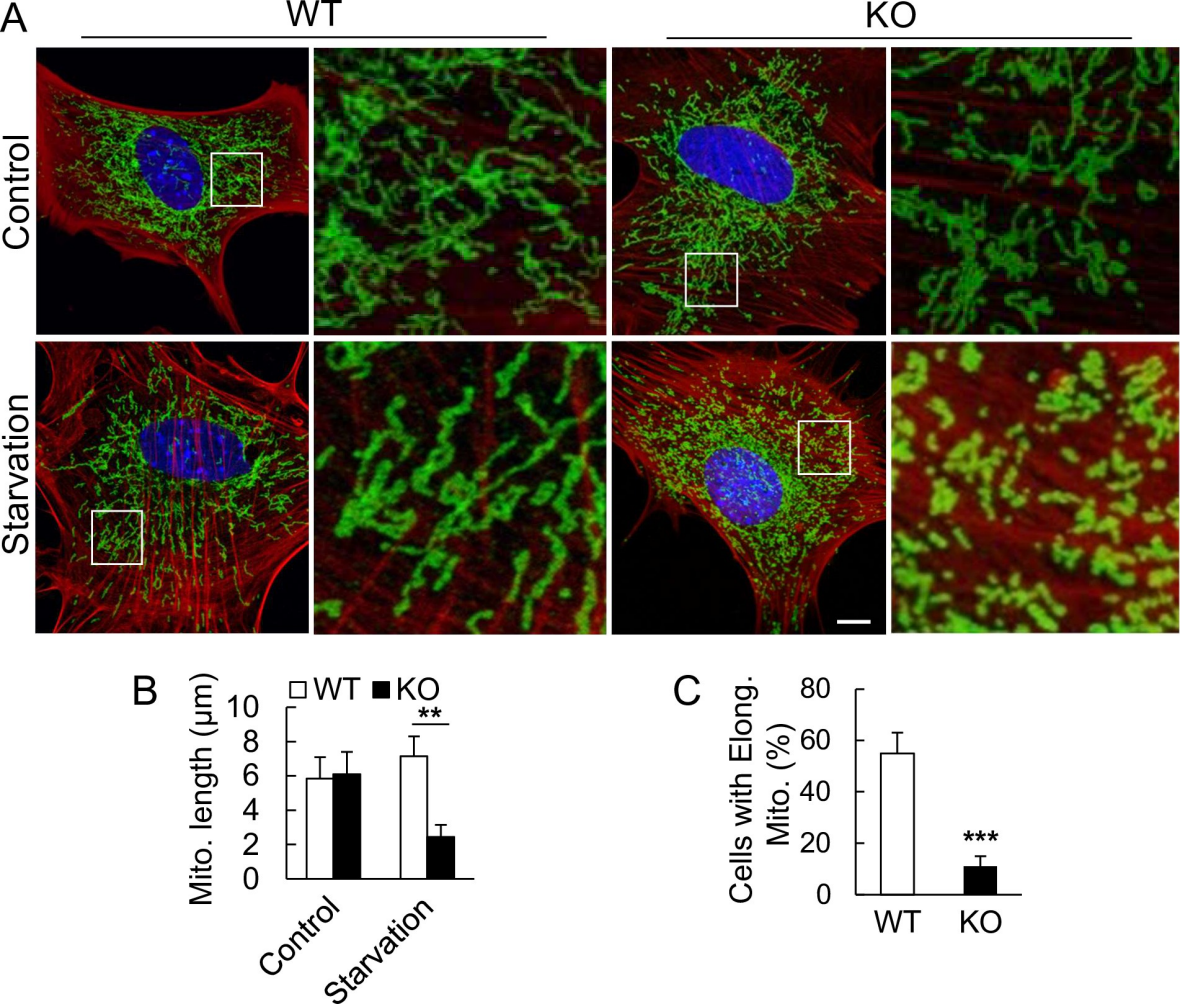
Lack of Ubl4A lead to mitochondrial fragmentation under nutrition deprivation. (A) Morphology of mitochondria in primary WT and KO MEFs under HBSS starvation conditions for 2 h. Mitochondria were immunostained with anti-Tom20 antibody and imaged with confocal fluorescence microscopy; phalloidin-stained actin was used to indicate the cell boundary. Bar, 10 μm. (B) Mitochondrial length analysis based on (A). ** p < 0.01. (C) Quantification of cells with elongated mitochondria (> 5 μm) under starvation. A total of 15 cells for each group was analyzed; *** p < 0.001.

To further compare the mitochondrial length between WT and KO quantitatively, we analyzed the mitochondria in early-passaged WT and KO mouse embryonic fibroblasts (MEFs). We found that, under non-starvation condition, both mitochondrial morphology and length were similar. However, upon starvation, the mitochondria in the Ubl4A KO cells were fragmented (Fig 2A) and significantly shorter (Fig 2B), and the number of cells with elongated mitochondria was also significantly lower (Fig 2C) than their WT controls. Together with the results of cell death phenotype (Fig 1), these results imply that mitochondrial fragmentation might disrupt mitochondrial function and contribute to the starvation-induced cell death in Ubl4A-deficient cells.

### Fragmented mitochondria in Ubl4A-deficient cells are mainly due to the failure of the fusion process

**Fig 3:**
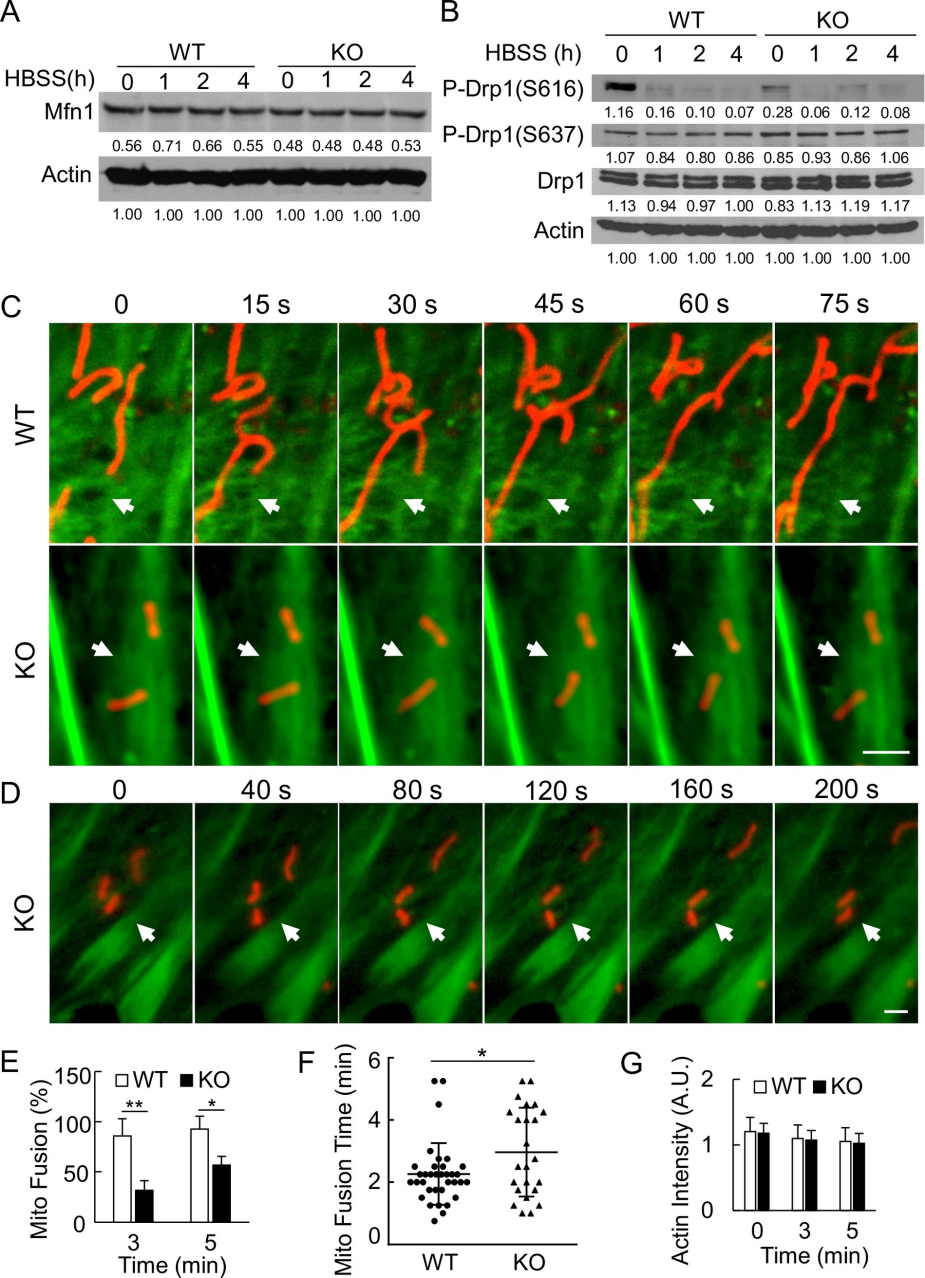
Fragmented mitochondria in Ubl4A-deficient cells are mainly due to the failure of the fusion process. (A) Immunoblotting of Mfn1 protein levels in MEFs under HBSS starvation for the indicated times. The intensity of each band was quantitated by ImageJ using actin band as the reference. (B) Immunoblotting with anti-p-Drp1 (S616), anti-p-Drp1 (S637) and anti-Drp1 antibodies in the WT and KO MEFs under starvation for indicated time. Actin was used as a protein loading control. (C) Time-lapse confocal images of WT and KO MEFs co-transfected with mEGFP-Lifeact-7 (green) and DsRed2-Mito-7 (red) under HBSS starvation conditions. A 6-min video of each cell was captured in 15-sec intervals; n = 15 for each group. Bar, 2 μm. (D) Extended time-lapse confocal images of KO MEFs with an interval of 40 sec. Bar, 2 μm. (E) Quantification of the percentage of fusion event for mitochondria in close proximity (< 2 μm) at indicated time points; * p < 0.05 and ** p < 0.01. (F) Non-parametric quantification of mitochondrial fusion events occurred during video acquisition. Seven events per cell for a total of 10 cells in each group were analyzed; * p < 0.05. (G) Quantification of actin fluorescent intensity. Ten areas with similar mitochondrial fluorescent intensities per cell for a total of 10 cells in each group were analyzed.

Mitochondrial dynamics are maintained by fusion and fission processes, and such fragmented mitochondria observed above could be derived from either defective fusion or enhanced fission. To examine this, we analyzed the level of mitofusin 1 (Mfn1) and the activity of dynamin-related protein 1 (Drp1), which are well-studied mitochondrial fusion and fission regulators, respectively. Mfn1 levels appeared steady throughout the starvation period (Fig 3A). Both phosphorylation levels of Drp1 S616 (a positive regulator of fission) [8], and Drp1 S637 (a negative regulator of fission) [9] appeared to remain steady during the starvation period (Fig 3B). Although the basal level of p-Drp1 S616 in the WT was higher than that in the KO, and the level of p-Drp1 (S637) over time course seemed slightly higher in the KO cells than in the WT cells, there were no obvious changes of Drp1 activities. Together, it appears that the fragmentation of mitochondria under starvation condition is not predominantly contributed by Drp1-mediated fission.

To further visualize the mitochondrial fusion process, we transfected both WT and Ubl4A KO cells with the mitochondrial marker DsRed2-Mito-7 and actin marker mEGFP-Lifeact-7, and subjected the cells to live time-lapse spinning-disc confocal microscopy. As shown in the time course images (Fig 3C, upper panel), the fusion process of mitochondria in the WT cells was directional and rapid (within 30 sec). However, the mitochondria in the Ubl4A KO cells appeared to move randomly and slowly with a difficulty reaching each other (Fig 3C, lower panel) even when they were in a close proximity for a longer period of time (Fig 3D). The results from the quantitative analysis showed that the population of successfully fused mitochondria in these Ubl4A KO cells was significantly less than that in the WT cells (Fig 3E), and the mitochondria in the Ubl4A KO cells required more time to finish the fusion process (Fig 3F). Of note, the overall level of actin density and assembly in both WT and KO cells were similar and no significant change during experimental time (Fig 3G), indicating the major actin network seems not affected by deficiency of Ubl4A. These results suggest that Ubl4A is involved in the mitochondrial fusion process in response to starvation insult.

### The actin-related protein Arp2/3 complex is critical for the mitochondrial fusion process under starvation conditions

It has been well documented that, under non-starvation conditions, inhibition of either actin or Arp2/3 results in an increased subpopulation of elongated mitochondria [20,30–32]. The underlying mechanism is that the disruption of the actin assembly led to release of mitochondria for fusion [20,30,31]. Furthermore, actin polymerization and constriction also plays a key role at ER-mitochondria contact sites in promoting fission process itself [31–36]. Although we did not observe obvious changes in overall actin intensity in both WT and KO cells upon starvation (Fig 3G), we wondered that whether the role of actin-branches and the Arp2/3 complex on mitochondria was the same under both starvation and non-starvation conditions.

**Fig 4:**
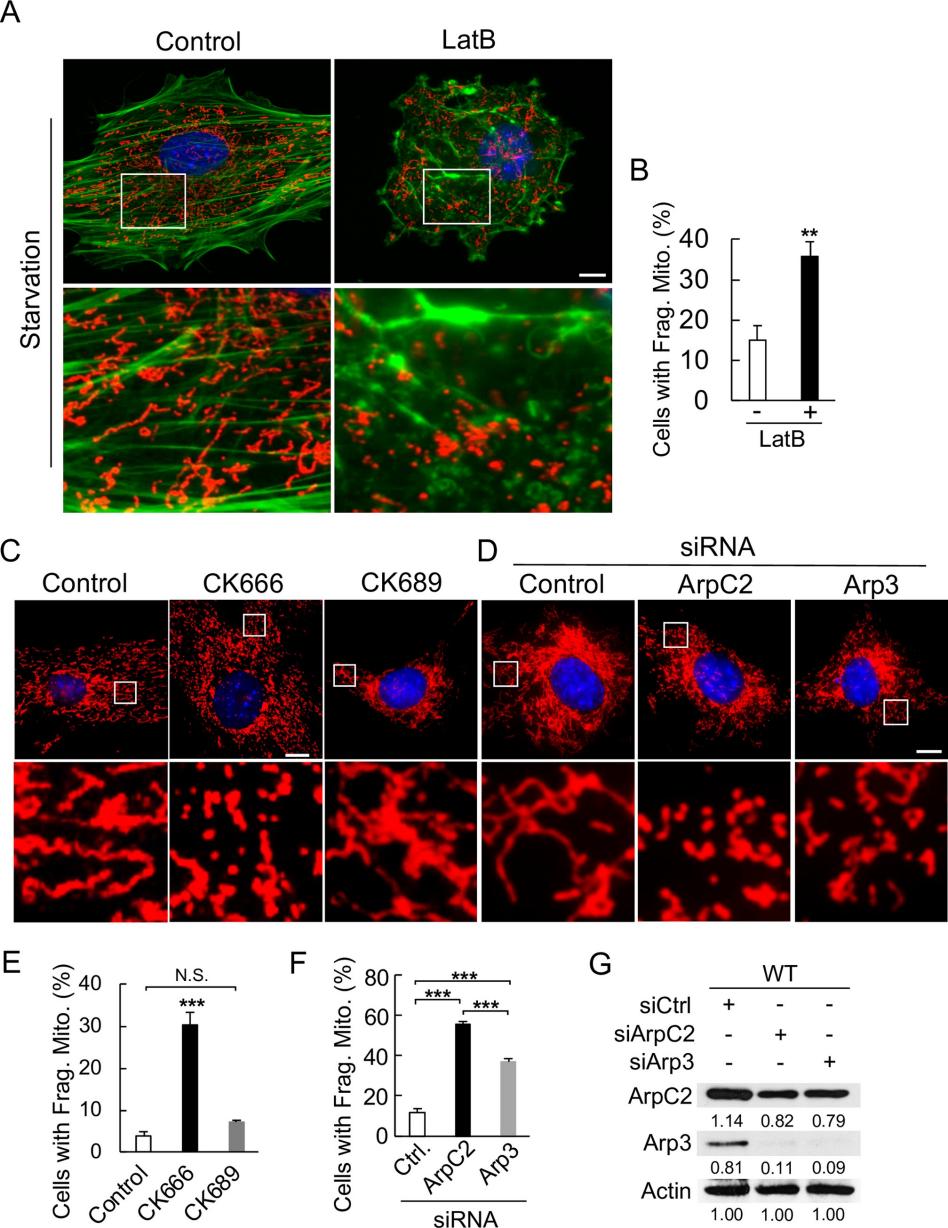
The actin-related protein Arp2/3 complex is critical for the mitochondrial fusion process under starvation conditions. (A) Morphology of mitochondria in the WT MEFs under HBSS starvation for 1.5 h with or without actin polymerization inhibitor latrunculin B (LatB, 10 nM). Mitochondria were immunostained with anti-Tom20 antibody, and actin was visualized with phalloidin. (B) Quantification of the cells in (A) with fragmented mitochondria. A total of 25 cells for each group were analyzed; ** p < 0.01. (C) Morphology of mitochondria in the WT MEFs under HBSS starvation for 2 h with or without the Arp2/3 inhibitor CK666 (30 μM). Mitochondria were visualized with MitoTracker Red. CK689 serves as an inactive control. (D) Morphology of mitochondria in the WT MEFs transfected with indicated siRNAs (control, mock siRNA) followed by starvation in HBSS for 2 h. Mitochondria were visualized with MitoTracker Red. (E) Quantification of the cells in (C) with fragmented mitochondria. A total of 25 cells for each group were analyzed; *** p < 0.001. (F) Quantification of cells from (D) with fragmented mitochondria. A total of 15 cells for each group were analyzed; ** p < 0.01 and *** p < 0.001. (G) Immunoblotting of ArpC2 and Arp3 in the WT MEFs from (D). Bar, 10 μm.

To test this, we pretreated WT MEFs with actin or Arp2/3 inhibitor and the length of mitochondria was monitored 2 h post-starvation. We found that the interruption of actin network significantly disables mitochondrial ability to maintain their length upon starvation. This resulted in a large population of fragmented mitochondria even at a low dose (10 nM) with a short starvation time (1.5 h) (Fig 4A and 4B). Similarly, treatment with Arp2/3 inhibitor CK666 (CK689 as an inactive control) also significantly increased the fragmentation of the mitochondria upon starvation stress (Fig 4C and 4E). To confirm that such defect was due to Arp2/3 complex, we used siRNAs against Arp2/3 individual components (Fig 4D). Similar to CK666, knocking down Arp2/3 with siRNAs against either ArpC2 or Arp3 also significantly increased the cell population with fragmented mitochondria (Fig 4F), although the protein levels of both ArpC2 and Arp3 were only slightly decreased (Fig 4G). Functionally, knocking down ArpC2 seemed more effective on the mitochondria than knocking down Arp3 (Fig 4G). These results suggest that the Ubl4A-mediated Arp2/3 function might be critical for the mitochondrial fusion process under starvation.

### Lack of Ubl4A results in a smaller pool of primed Arp2/3 surrounding mitochondria

**Fig 5:**
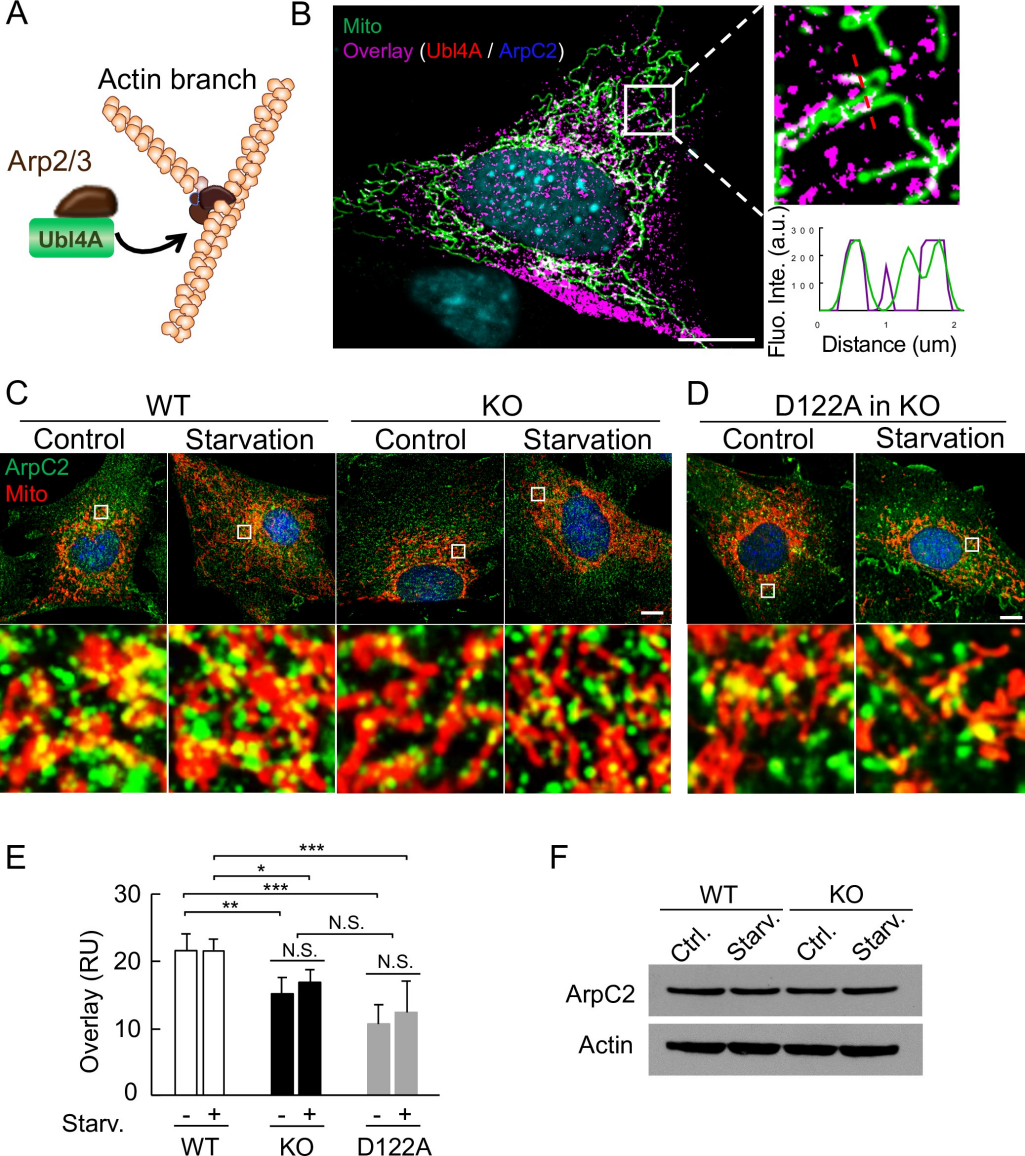
Lack of Ubl4A results in a smaller pool of primed Arp2/3 surrounding mitochondria. (A) Schematic representation (not to scale) of the relationship among Ubl4A, the Arp2/3 complex and actin Y-shaped branch. (B) Localization of endogenous Ubl4A, ArpC2 and mitochondria in WT MEFs. The cells were transfected with DsRed2-Mito-7 for 16 h, and then immunostained with anti-Ubl4A and anti-ArpC2 antibodies. Colocalized proteins (pseudocolored white) among Ubl4A (red), ArpC2 (blue) and mitochondria (green) were shown in the enlarged image. The overlay (magenta) of Ubl4A and ArpC2 was extracted to eliminate the background, based on binary image analysis. Line scan of the fluorescent intensity (insert). Bar, 10 μm. (C) and (D) Colocalization of endogenous ArpC2 and mitochondria in WT and KO (C), and KO MEFs transfected with D122A (D). Cells were also transfected with DsRed2-Mito-7 for 16 h and then immunostained with anti-ArpC2 antibody (green). Bar, 10 μm. (E) Quantitation of overlay fluorescent intensities of ArpC2 and mitochondria in selected regions with similar mitochondrial densities based on (C) and (D). Ten fields from a total of 10 cells for each group were analyzed; * p < 0.05 and ** p < 0.01. (F) Immunoblotting of ArpC2 protein in cells shown in (C). Actin was used as a protein loading control.

We previously reported that Ubl4A directly interacts with the actin-related protein (Arp2/3) complex to accelerate actin branching process (Fig 5A) [49]. To further examine Ubl4A and Arp2/3 in relation to mitochondria under starvation stress, we evaluated the colocalization of these three components. To avoid the background noise, we removed free Ubl4A and Arp2/3 staining to specifically focus on the spatial relationship between Ubl4A-Arp2/3 and mitochondria. We found that Ubl4A-Arp2/3 could be easily detected in colocalization with mitochondria (Fig 5B, white color), especially in the regions nearby the nucleus. As Arp2/3 is known to be critical for mitochondrial dynamic processes, we were wondering whether the distribution of Arp2/3 around the mitochondria was affected by Ubl4A deficiency. To test this, we coimmunostained ArpC2 with a mitochondrial marker (Fig 5C) and quantitatively compared the cellular overlay of Arp2/3 and mitochondria (Fig 5E) for both WT and Ubl4A KO cells under starvation conditions. The results show that the immunostaining overlay of Arp2/3-mitochondria was similar prior- and post-nutrient starvation within the individual groups for both WT and Ubl4A KO cells. However, to our surprise, the overlay of the Arp2/3-mitochondria in the Ubl4A KO cells was significantly less compared with the WT cells, regardless of starvation or not (Fig 5E), although the total Arp2/3 protein levels were similar in both cell groups (Fig 5F). Furthermore, the KO cells expressing the Arp2/3 binding-deficient Ubl4A mutant (D122A) [50] also had a significant less overlay (Fig 5D and 5E). These results indicate that Ubl4A is critical for gathering the Arp2/3 complex nearby mitochondria as “ready-to-go” pool; without this primed Arp2/3 complex nearby, the mitochondria are unable to fuse fast enough to sustain the starvation stress.

### Ubl4A WT, but not Arp2/3-binding deficient mutant, can rescue mitochondria-fragmentation phenotype under starvation condition

**Fig 6:**
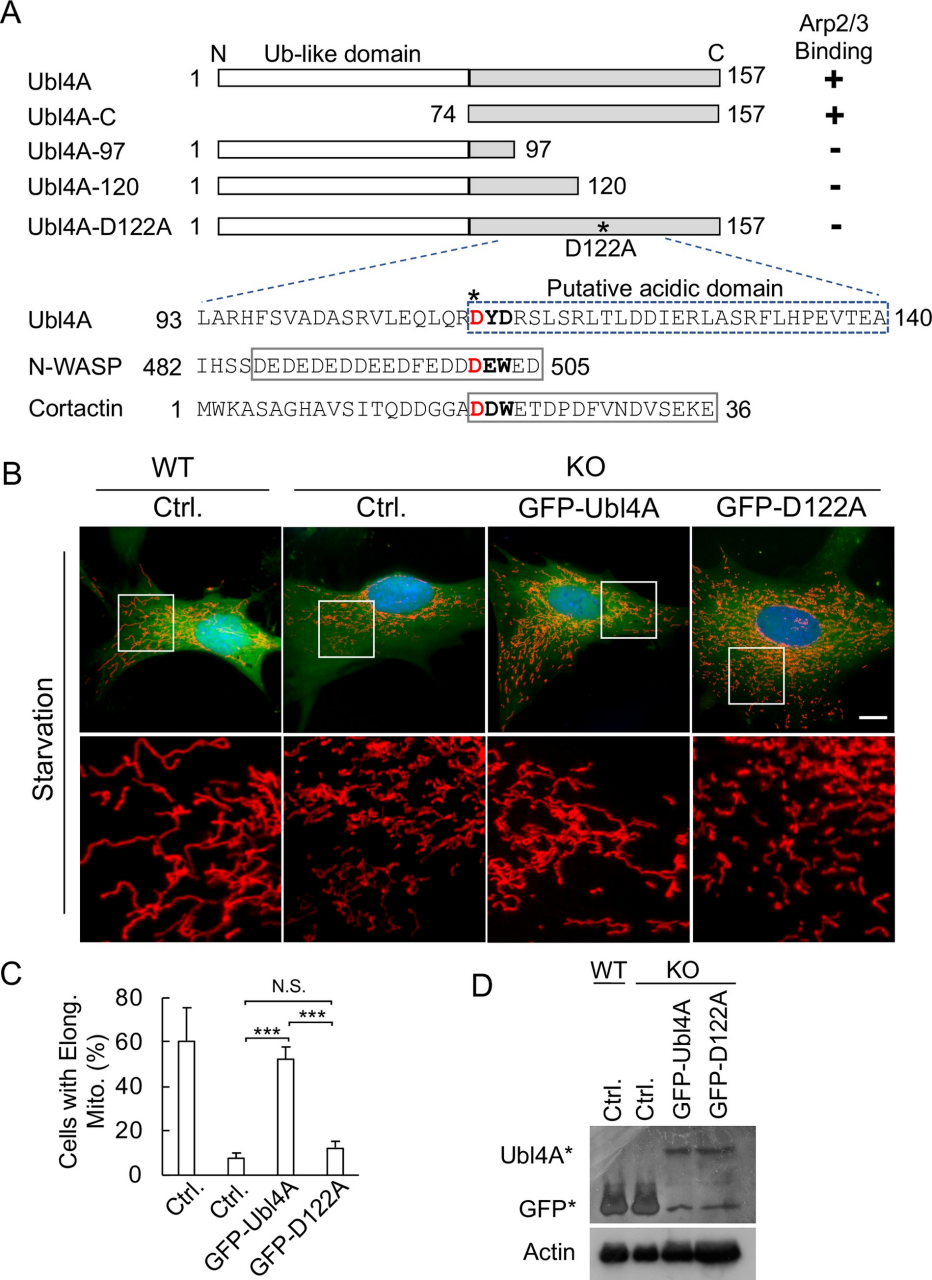
Ubl4A WT, but not Arp2/3-binding deficient mutant, can rescue mitochondria-fragmentation phenotype under starvation condition. (A) Schematic representation of WT Ubl4A and 4 mutant constructs (not to scale) and their abilities to bind the Arp2/3 complex. Enlarged peptide sequences are shown. The conserved “DYD” Arp2/3-binding domain is aligned with N-WASP and cortactin sequences. The acidic domains in N-WASP and cortactin are in boxes with solid lines, and the putative acidic domain in Ubl4A is in a box with dash lines. (B) Rescue assay in WT and KO MEFs transfected with GFP-tagged Ubl4A or D122A constructs for 16 h and starved in HBSS for 2 h. Ctrl, GFP only. Mitochondria were visualized with MitoTracker Red. Enlarged boxed regions shown below. Bar, 10 μm. (C) Quantification of cells with elongated mitochondria as shown in (B). A total of 15 cells for each group was analyzed; *** p < 0.001. (D) Immunoblotting with anti-GFP antibody of cells from (B). The upper bands (Ubl4A*) represent full-length constructs, and the lower bands (GFP*) represent degraded GFP proteins.

To confirm the hypothesis that the primed Arp2/3 pool around mitochondria was controlled by Ubl4A, we performed rescue experiments. We have shown that Ubl4A directly binds to Arp2/3 through its C-terminus, which contains a putative acidic domain conserved in several Arp2/3 binding partners, such as N-WASP and cortactin (Fig 6A) [52–54]. A single point mutation D122A diminished the ability of Ubl4A to bind Arp2/3 [50]. For the rescue experiment, we transfected either WT or Arp2/3-binding deficient Ubl4A (D122A) construct into KO cells. After starvation, we found that the mitochondria in the control group cells fragmented as expected; and the expression of WT Ubl4A into these KO cells partially rescued the mitochondria from fragmentation upon starvation. However, the expression of Ubl4A D122A mutant in the KO cells failed to prevent mitochondria from fragmentation (Fig 6B and 6C), although the mutant protein was expressed at a level similar to that of the WT (Fig 6D). These results indicate that the association of Ubl4A with Arp2/3 may be required for mitochondria to quickly fuse in response to nutrient deprivation insult.

### Ubl4A-deficient mediated cell death under starvation stress is through mitochondrial dependent apoptosis

**Fig 7:**
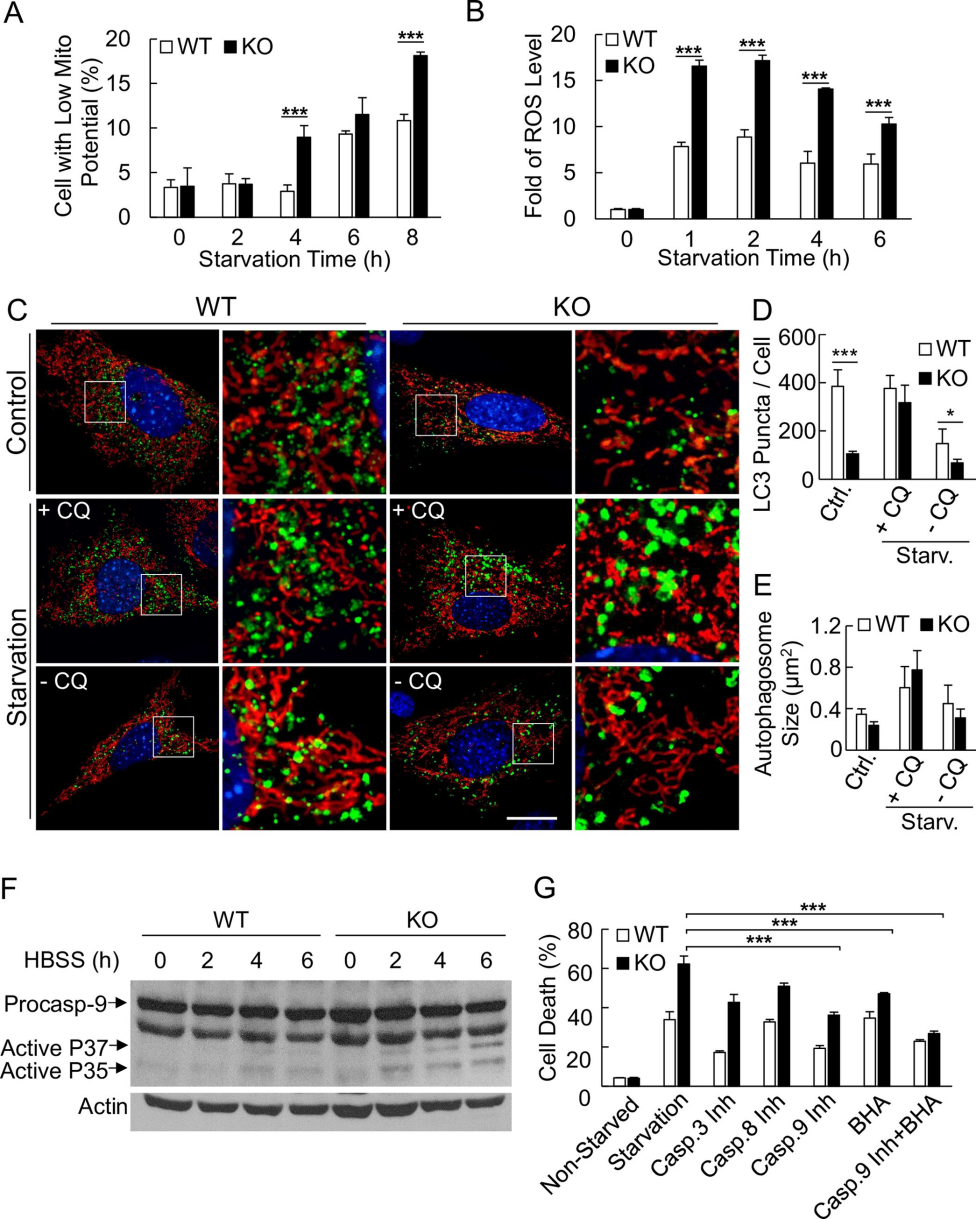
Ubl4A-deficient mediated cell death under starvation stress is through mitochondrial dependent apoptosis. (A) Mitochondrial transmembrane potential assay. WT and KO MEFs were starved in HBSS for the indicated time. The cells were stained with TMRE and subjected to flow cytometry analysis. (B) Cellular ROS level assay. WT and KO MEFs were in HBSS for indicated times. The cells were stained with H2DCF and subjected to flow cytometry analysis. The value represents the fold difference compared to the level of non-starved cells at the “0” time point for each group. All experiments were repeated independently at least three times for statistical analysis; *** p < 0.001. (C) Visualizations of LC3 puncta and mitochondria in WT and KO MEFs cells that co-transfected with GFP-LC3 and DsRed2-Mito-7 treated with or without chloroquine (CQ, 30 μM) under normal or starvation condition. (D) Quantification of LC3 puncta number per cell based on cells from (C); * p < 0.05 and *** p < 0.001. (E) Quantification of LC3 puncta sizes in cells from (C). Both quantification analysis were performed using ImageJ with the particle analysis function based on binary images. The threshold was set to exclude out particles that smaller than 0.01 μm. Seven whole cell images from each group were analyzed. (F) Immunoblotting of caspase 9 in the WT and KO MEFs under starvation for the indicated time; actin was used as a protein-loading control. (G) Cell death assay under HBSS starvation for 16 h with or without the indicated inhibitors (Inh, inhibitor; BHA, butylated hydroxyanisole).

In the above experiments, we have shown that deficiency in Ubl4A caused mitochondrial fragmentation under starvation conditions. We wondered whether such fragmented mitochondria could maintain their integrity and function. If not, whether the cell death is through apoptosis or autophagy. To test this, we carefully examined mitochondrial functional status during a time course of starvation. We found that, upon starvation insult, mitochondrial membrane potential in the Ubl4A KO cells dropped faster and cells with low mitochondrial potential were spikily surged at earlier hours (Fig 7A) and the cellular ROS level significantly accumulated in less than one hour upon starvation (Fig 7B). Interestingly, when we looked into autophagy, we found that under non-starvation condition, the Ubl4A KO cells had less LC3- puncta (Fig 7C and 7D). However, upon starvation, both WT and KO cells responded in a similar fashion, although KO cells had moderate smaller number of LC3-autophgosomes but in larger sizes (Fig 7D and 7E). These date indicate that Ubl4A-deficient cells seemed able to form autophagosome similarly to the WT cells. However, the loss of mitochondrial integrity at the early stage of starvation might trigger the release of cytochrome C and the activation of mitochondrial cell death. Indeed, the activation of caspase 9 (cleaved P35 and P37) could be detected around 2 h post-starvation by immunoblotting (Fig 7F), and inhibition of both caspase 9 and ROS appeared to be more effective in blocking starvation-induced cell death (Fig 7G, Casp 9 inh + BHA). These results further evidenced that the delay of Ubl4A/Arp2/3-mediated mitochondrial fusion led to the loss of mitochondrial integrity, consequently initiated mitochondria-dependent apoptotic cell death before autophagic rescue.

In summary, here we demonstrated that the Arp2/3-actin network plays a critical role in mitochondrial fusion process and cell survival under starvation conditions. This seems institutively contradict with the current theory that Arp2/3-actin plays a key role in mitochondrial fission. It is possible that differential roles of actin network could be co-exist or dictated by different cell types or cellular context in response to specific microenvironmental stimuli. In response to starvation insult, this group of mitochondria could respond quickly and fuse in a timely fashion to conserve energy and avoid triggering cell death. Lack of Ubl4A, the mitochondrial fusion process could be delayed and fragmented mitochondria accumulate and activate apoptosis at the early stage of starvation before autophagy kicked in for cell survival.

## Discussion

Ubl4A is a multifunctional protein that is involved in diverse cellular events, from translocation of newly synthesized proteins to the ER, DNA damage repair, bone modeling, inflammatory and innate immune response, to anti-tumorigenesis [40–48]. Here, we showed that under nutrient starvation conditions, Ubl4A functions as a survival factor mainly by regulating the mitochondrial fusion process. Such function appears to be dependent on the Arp2/3 complex. It is well-known that the Arp2/3-actin network controls mitochondrial fission process [20,30–32]. Under non-starvation conditions, breakdown of the actin microfilament causes the release of mitochondria, enabling them to fuse [20,30,31]. In addition, actin polymerization and contraction at ER-mitochondrial contacting sites also play a key role in promoting mitochondrial fission itself [31–36]. However, the function of Arp2/3-actin in mitochondrial fusion process in mammalian cells still remains to be explored. It has been shown that the Arp2/3-actin is critical for mitochondrial mobility in budding yeast and mutations of Arp2/3 subunit led to loss of all directed movement and defects in mitochondrial morphology [26–29]. To our knowledge, this is the first report that Arp2/3-actin is critical for mitochondrial fusion and subsequently for cell survival under starvation condition in mammalian cells.

The function of Arp2/3-actin in mitochondrial fusion appears to rely on Ubl4A, which “holds” the Arp2/3 complex nearby mitochondria serving as a “ready-to-go” pool. However, with sufficient nutrient supplies, such “ready-to-go” pool is not essential and can be compensated in Ubl4A deficient cells, as both WT and KO cell growth and mitochondrial status (number and length) are similar. On the other hand, under starvation conditions, this pool of primed Arp2/3 complex is vital for a quick response to starvation insult in facilitating the mitochondrial fusion process. Without it, cellular ROS levels quickly surge within an hour of starvation, followed by the loss of mitochondrial membrane potential, activation of caspase 9, and apoptotic cell death.

Nutrient starvation is closely associated with autophagy. The Arp2/3-mediated actin network is known to participate in the formation of autophagosomes [55,56]. Recent evidence has also shown that Ubl4A directly interacts with LAMP1 in the autophagosome-lysosome fusion process [40]. However, we did not observe any significant defect in autophagy under our experimental conditions. One explanation is that timing may be the critical factor here. The loss of mitochondrial membrane potential and accumulation of ROS occur so fast at the early stage of starvation process that there is not enough time for autophagy to be fully in place before cell death commences. Therefore, the initial delay in mitochondrial fusion could be a time-control factor that determines the fate of the cells in response to starvation stress.

As the majority of the Arp2/3 complex is known to bind actin [22–24], we used the Arp2/3 complex level as an indicator of a fine actin meshwork. The overall intensities of actin filament staining were similar in both wild-type and Ubl4A-deficient cells. However, due to the high amount of actin filaments in cells, it is difficult to visualize defects in the fine actin branches in Ubl4A-deficient cells without using a high-resolution image tool. Immuno-electronic microscopy may be needed to directly visualize such defects at the microfilament level. Whether the Arp2/3 complex can act on Ubl4A independently of actin branching networks for mitochondrial fusion remains an open question.

We have previously shown that Ubl4A directly interacts with the Arp2/3 complex to promote acting branching [49]. However, which subunit of Arp2/3 complex interacts with Ubl4A is still needed to be confirmed. We speculate that Ubl4A most likely binds Arp3 subunit from the following two reasons: 1) Co-immunoprecipitation with anti-Ubl4A antibody and *in vitro* pull-down assay with purified recombinant Ubl4A and Arp2/3 complex could bring down Arp3 [49]; 2) The Arp2/3 partners, N-WASP and cortactin, are well known to compete for binding to Arp3 through their conservative acidic domains [52–54]. Ubl4A also has a putative acidic domain and point mutation (D122A) in this domain could abolish the interaction between Ubl4A and Arp2/3 [50]. Therefore, it is most likely that Arp3 is the critical subunit which directly associates with Ubl4A. This conclusion is also consistent with the results from the functional rescue experiments. Ectopic expression of the wild-type Ubl4A, but not the Arp2/3-binding deficient mutant (D122A), rescued the mitochondrial fusion phenotype in the Ubl4A-deficient cells under starvation conditions. Logically, the put-back of Ubl4A should also rescue the KO cells from starvation-induced death. However, such reinstatement by ectopic expression of wild-type Ubl4A caused cell death [50]. This outcome is not surprising and consistent with the pro-death function of Ubl4A in anti-tumorigenesis [41]. We have demonstrated that the pro-death domain of Ubl4A is located in its C-terminal region [50], which is also critical for the interaction with the Arp2/3 complex [49,50]. Therefore, the pro-death function of Ubl4A appears to be dependent on its ability to bind the Arp2/3 complex, since Ubl4A-Arp2/3 binding deficient mutant D122A is unable to induce cell death [50]. However, we cannot exclude out the possibility that the D122A mutation might interrupt other functions of Ubl4A that are independent of its binding to Arp2/3. Furthermore, whether Ubl4A-induced cell death also relies on Arp2/3-dependent actin branching needs to be further explored.

Ubl4A proteins have been detected in association with several cellular organelles, such as the ER and nucleus [42,43,57]. We also detected some Ubl4A proteins in the mitochondria-rich fraction. Since Ubl4A can directly bind Arp2/3, it is possible that Ubl4A serves as an adaptor between mitochondria and the Arp2/3 complex to hold them in close proximity to facilitate mitochondrial fusion process. Defects in Ubl4A result in impaired mitochondrial ends towards each other and delaying mitochondrial fusion process. We also noticed that, even when two mitochondrial ends were in close proximity to each other, they still had a difficulty to finish the fusion process, indicating that the Ubl4A or the Ubl4A-associated Arp2/3 complex might also play a role in the mitochondrial outer membrane fusion itself. Whether Ubl4A promotes mitochondrial fusion through other indirect mechanism other than Arp2/3 also remains to be explored.

## Supporting information

S1 Raw images (PDF)
