# Supplementary figures and images for: Ubl4A is critical for mitochondrial fusion process under nutrient deprivation stress
Source: PLoS One. 2020 Nov 19;15(11):e0242700. doi: 10.1371/journal.pone.0242700 (PMC7676689; doi:10.1371/journal.pone.0242700)

Fig 3A

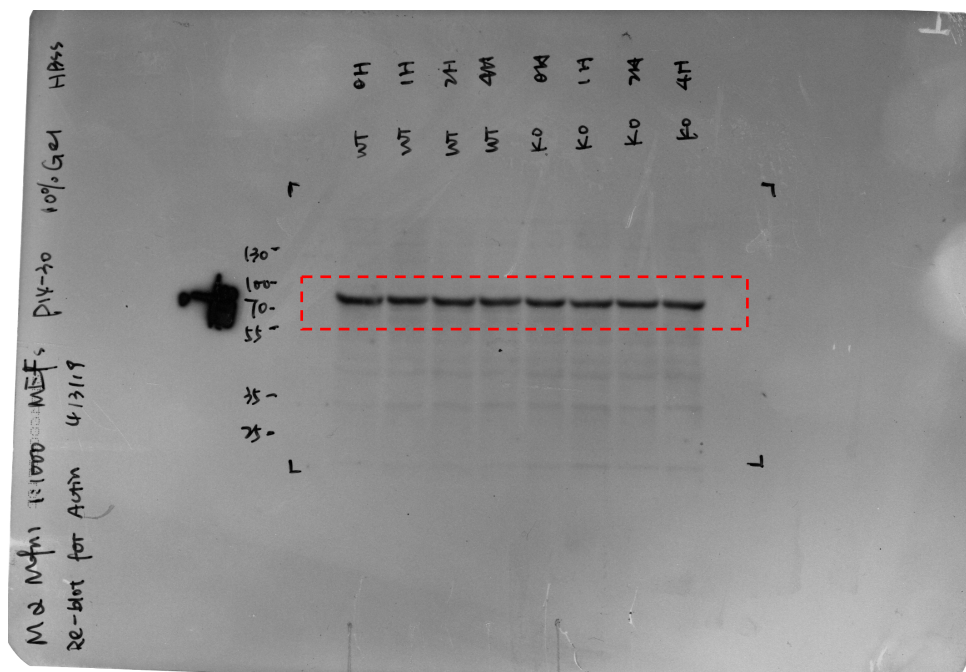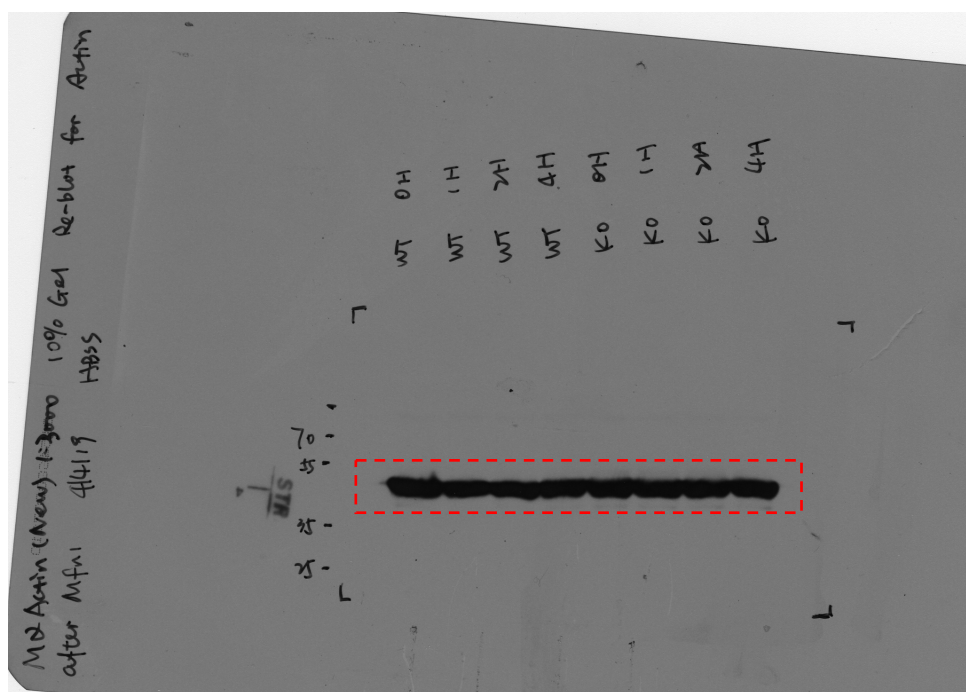

Fig 3B

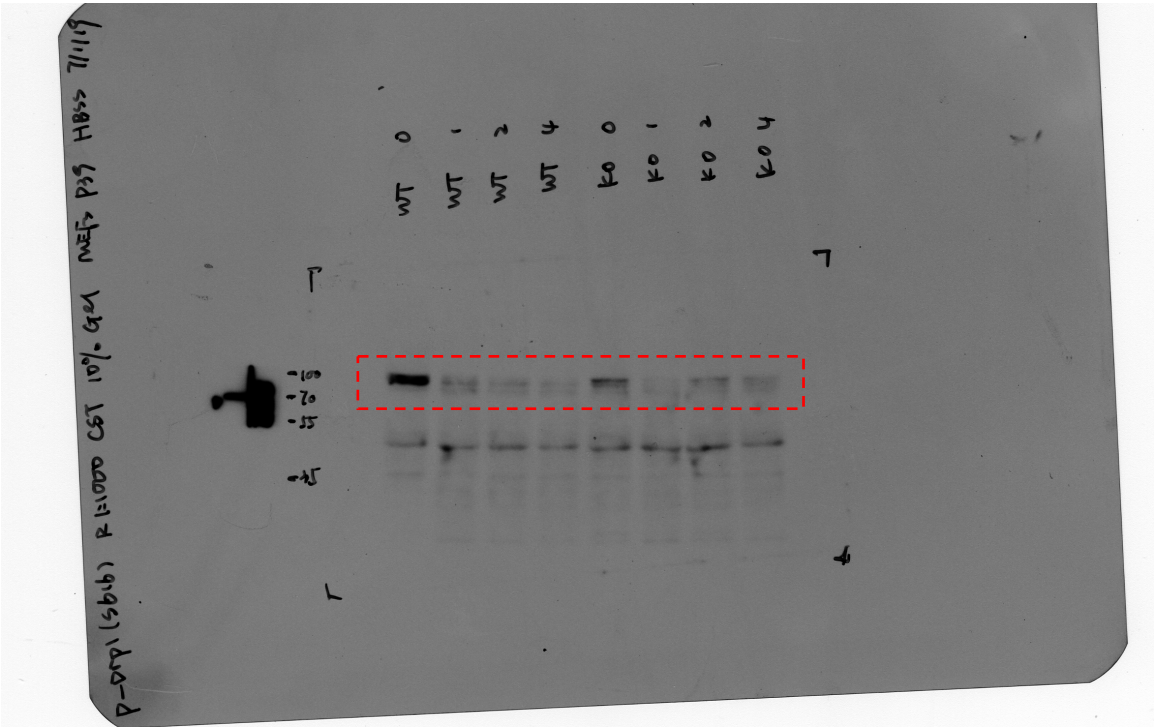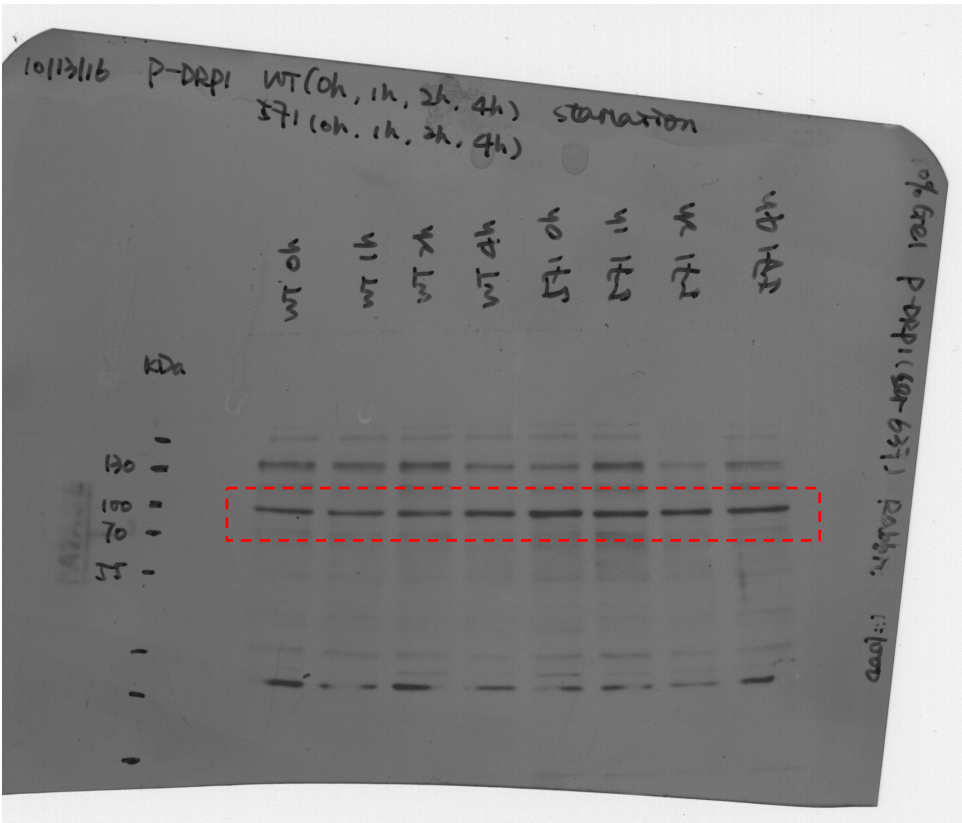

Fig 3B, continued

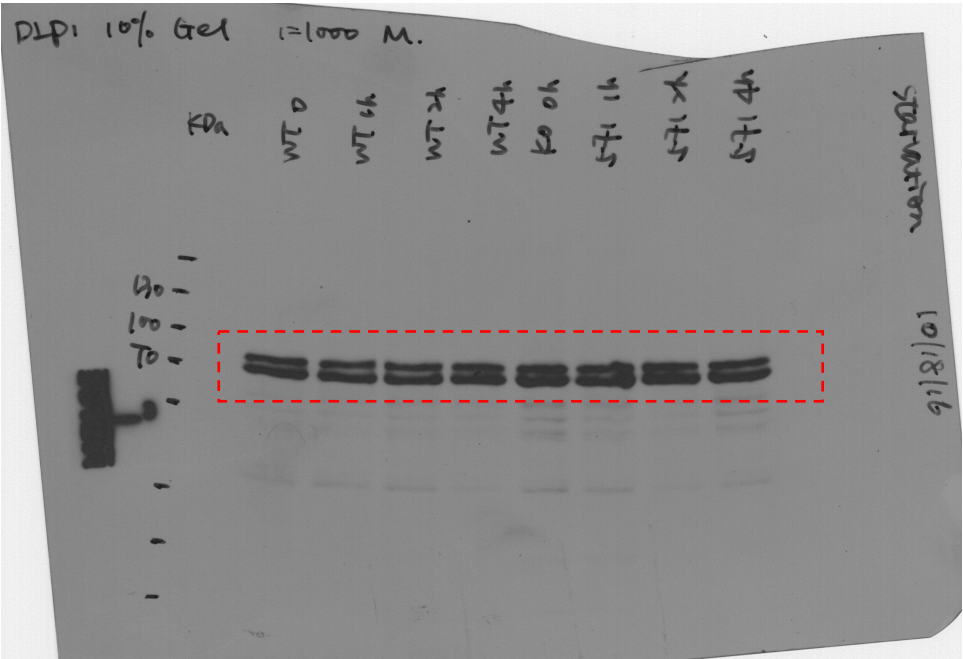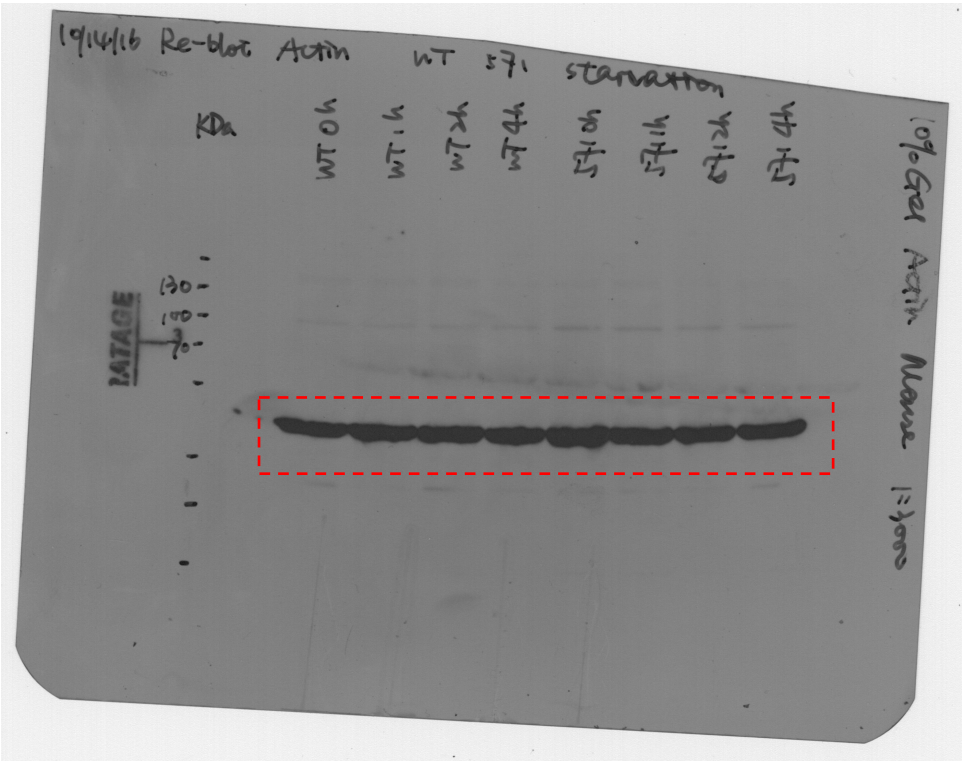

Fig 4G

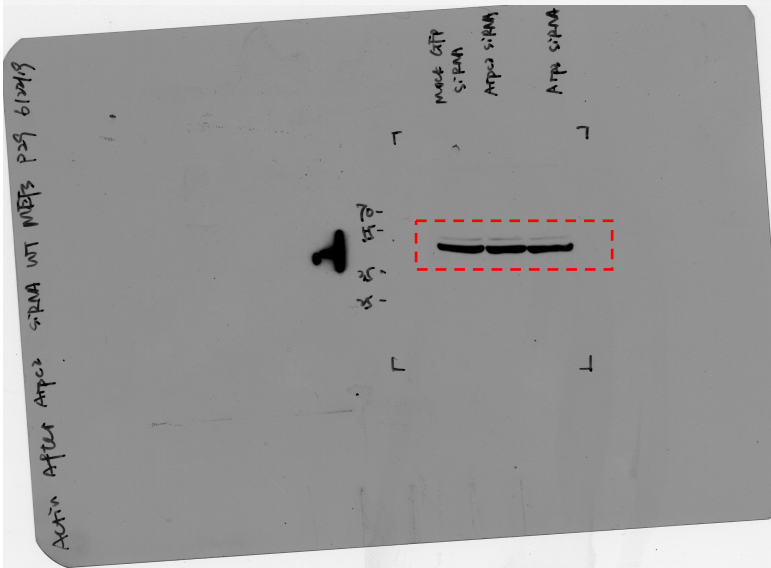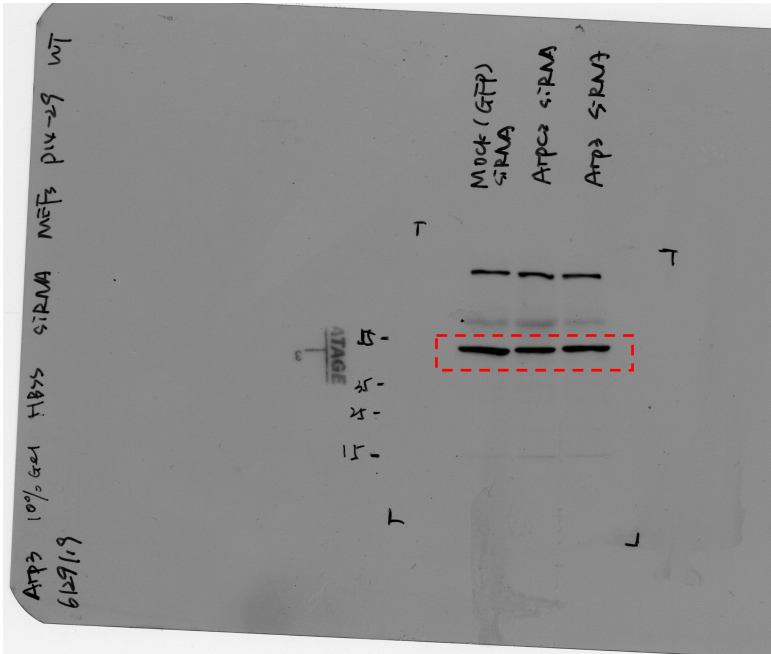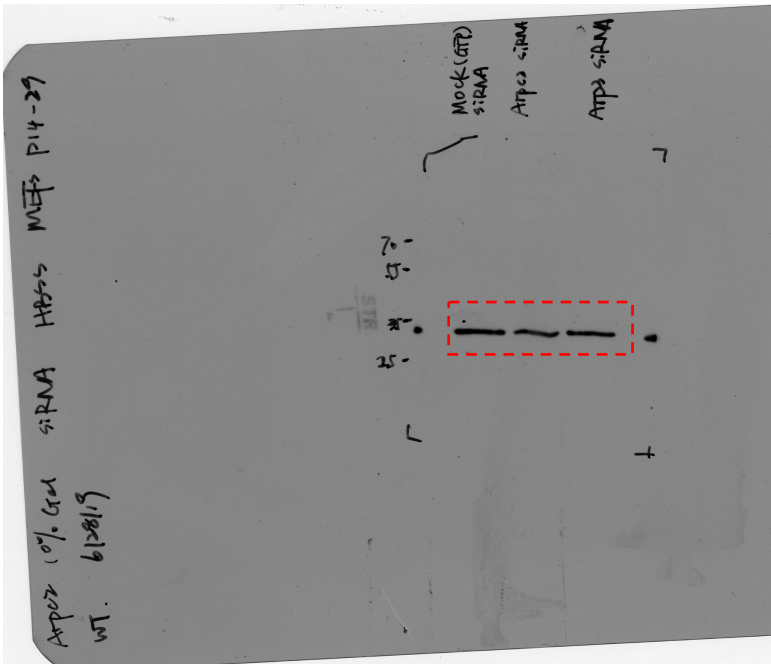

Fig 5E

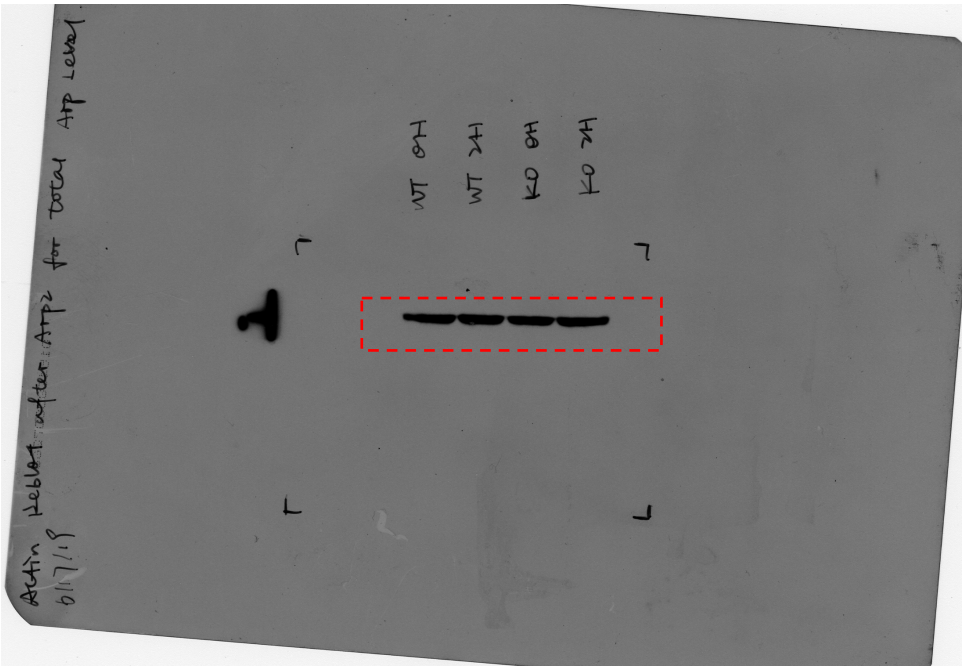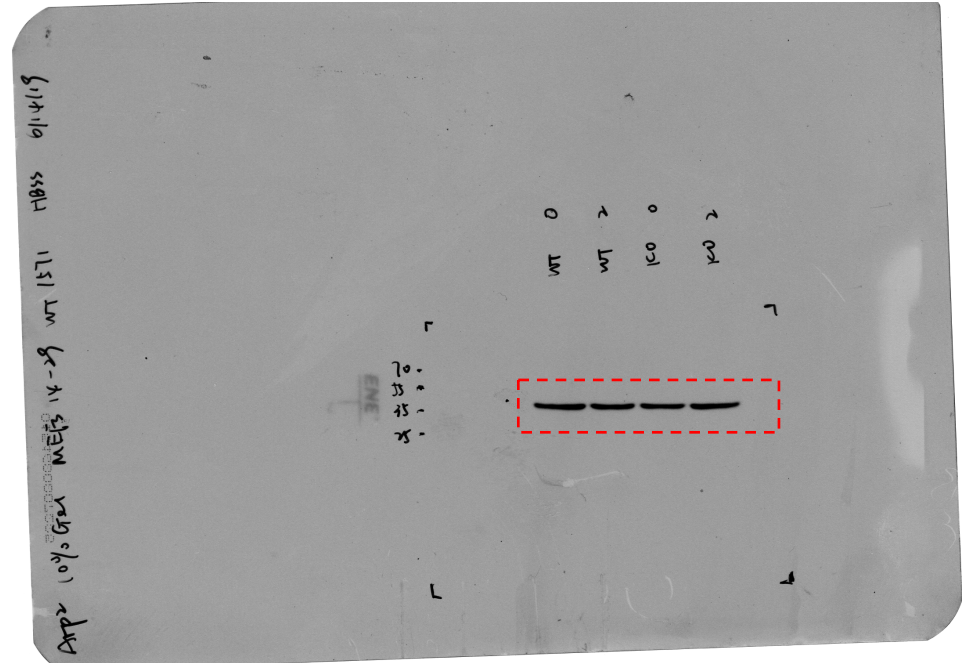

Fig 6D

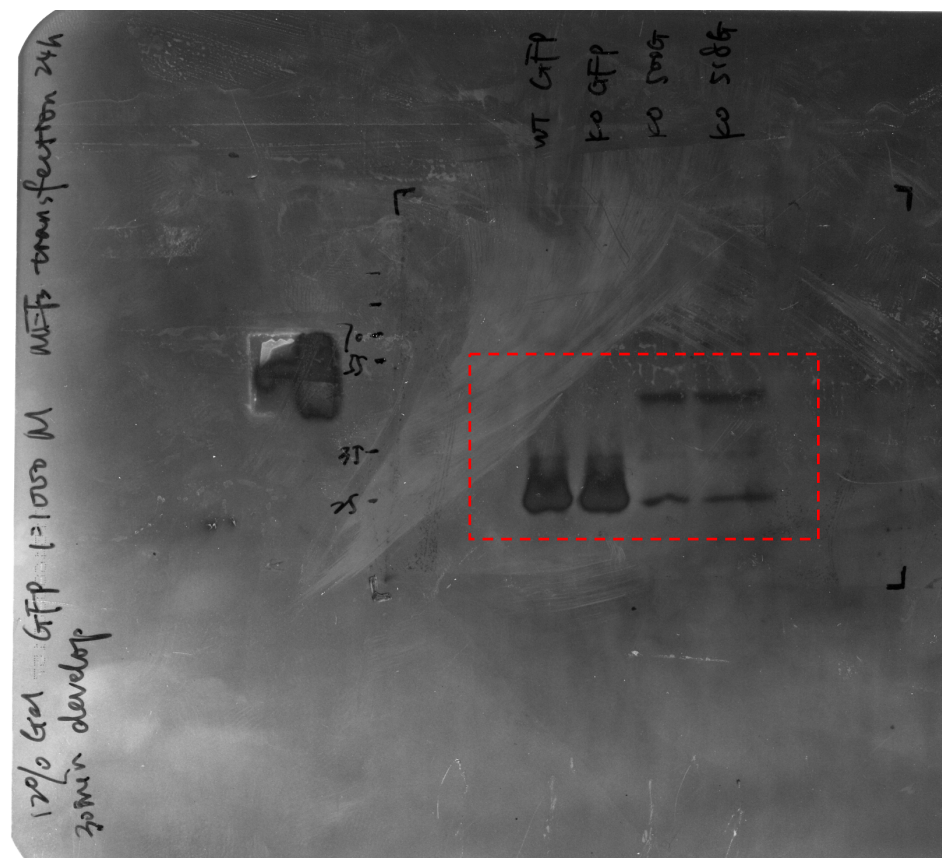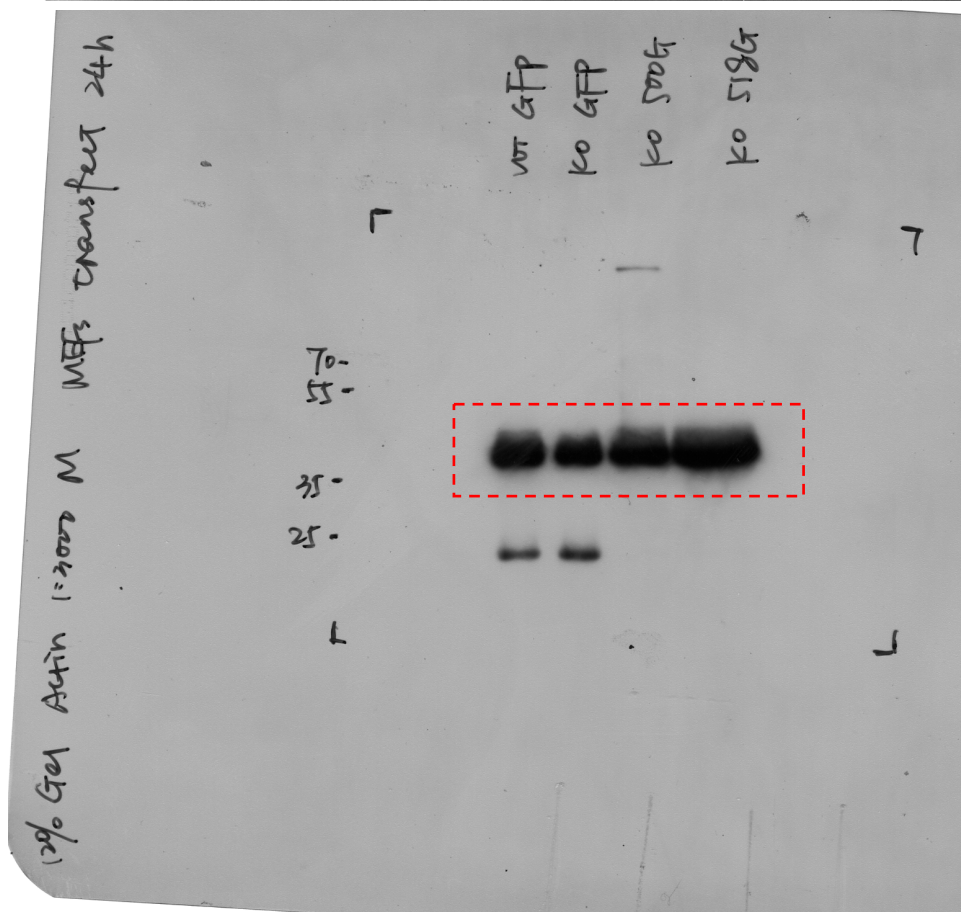

Fig 7F

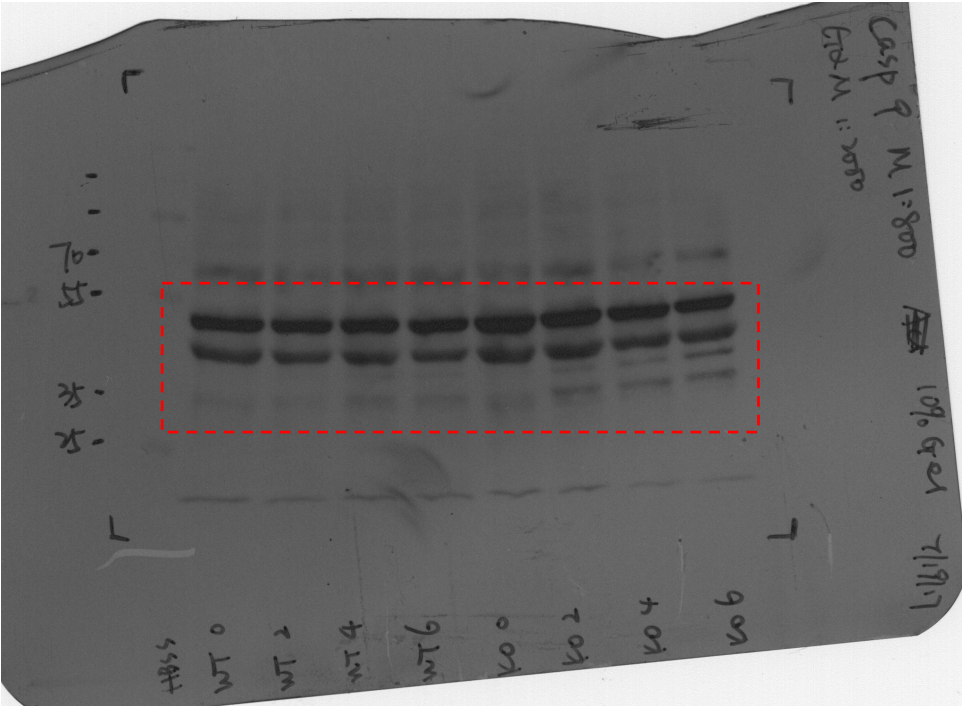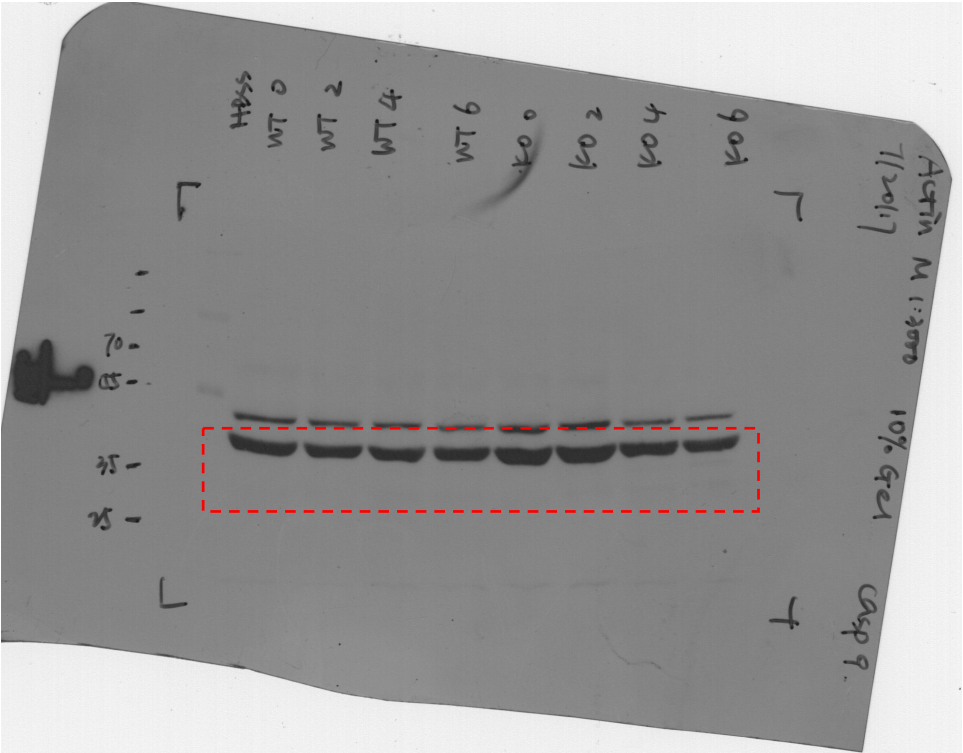

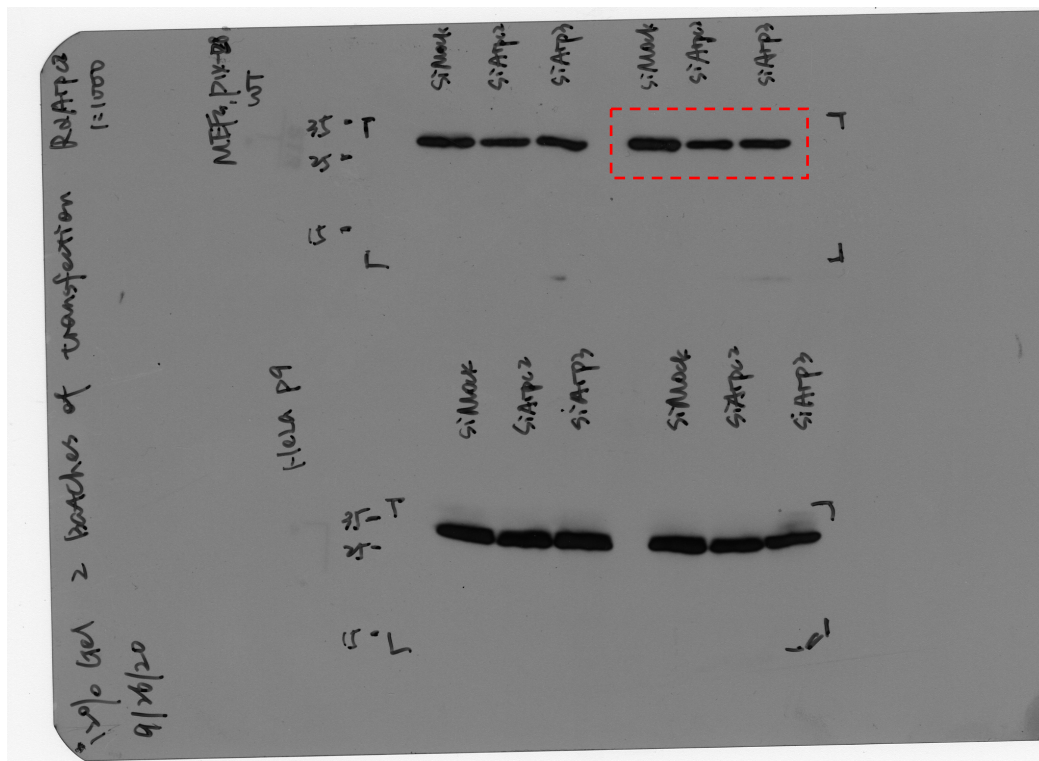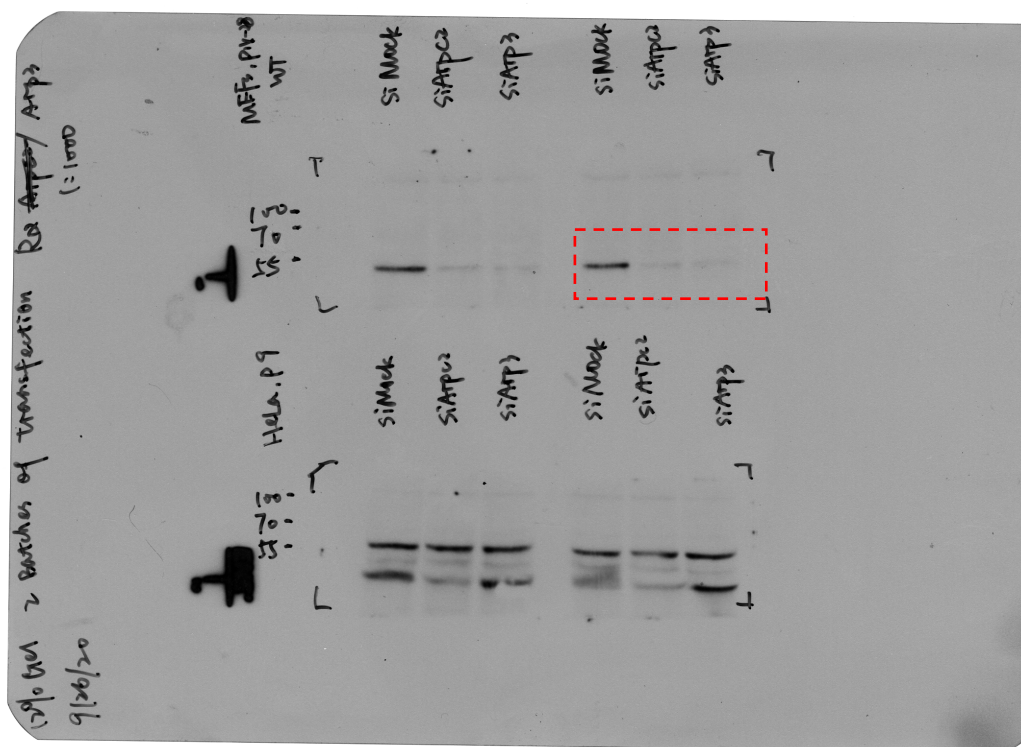

Revised Fig 4G, continued

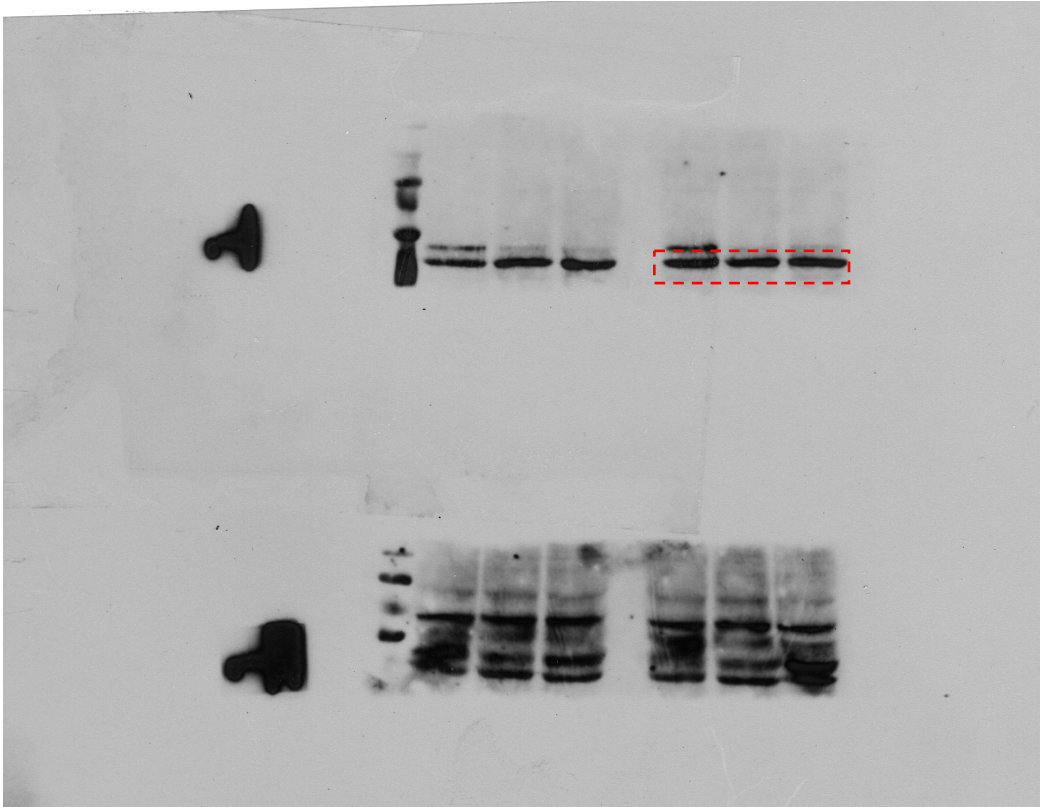

Supplement: S1 Raw images — (PDF) [file pone.0242700.s001.pdf]
